# Cell wall synthesis and remodelling dynamics determine division site architecture and cell shape in *Escherichia coli*

**DOI:** 10.1038/s41564-022-01210-z

**Published:** 2022-09-12

**Authors:** Paula P. Navarro, Andrea Vettiger, Virly Y. Ananda, Paula Montero Llopis, Christoph Allolio, Thomas G. Bernhardt, Luke H. Chao

**Affiliations:** 1 Department of Molecular Biology, Massachusetts General Hospital, Boston, MA USA; 2 Department of Genetics, Blavatnik Institute, Harvard Medical School, Boston, MA USA; 3 Department of Microbiology, Blavatnik Institute, Harvard Medical School, Boston, MA USA; 4 MicRoN Core, Harvard Medical School, Boston, MA USA; 5 Faculty of Mathematics and Physics, Mathematical Institute, Charles University, Prague, Czech Republic; 6 Howard Hughes Medical Institute, Harvard Medical School, Boston, MA USA

**Keywords:** Cellular microbiology, Bacterial development, Bacterial evolution, Cryoelectron tomography, Cell growth

## Abstract

The bacterial division apparatus catalyses the synthesis and remodelling of septal peptidoglycan (sPG) to build the cell wall layer that fortifies the daughter cell poles. Understanding of this essential process has been limited by the lack of native three-dimensional views of developing septa. Here, we apply state-of-the-art cryogenic electron tomography (cryo-ET) and fluorescence microscopy to visualize the division site architecture and sPG biogenesis dynamics of the Gram-negative bacterium *Escherichia coli*. We identify a wedge-like sPG structure that fortifies the ingrowing septum. Experiments with strains defective in sPG biogenesis revealed that the septal architecture and mode of division can be modified to more closely resemble that of other Gram-negative (*Caulobacter crescentus*) or Gram-positive (*Staphylococcus aureus*) bacteria, suggesting that a conserved mechanism underlies the formation of different septal morphologies. Finally, analysis of mutants impaired in amidase activation (Δ*envC* Δ*nlpD*) showed that cell wall remodelling affects the placement and stability of the cytokinetic ring. Taken together, our results support a model in which competition between the cell elongation and division machineries determines the shape of cell constrictions and the poles they form. They also highlight how the activity of the division system can be modulated to help generate the diverse array of shapes observed in the bacterial domain.

## Abstract (web summary)

In situ cryo-ET imaging and live-cell fluorescence microscopy reveal that septal peptidoglycan architecture and divisome activity modulate bacterial morphogenesis in *Escherichia coli*.

## Main

Bacterial cells are typically surrounded by a multi-layered cell envelope of varying complexity depending on species^1^. Gram-positive bacteria possess a single membrane surrounded by a thick cell wall, whereas Gram-negative bacteria have a thinner wall covered by an outer membrane (OM)^2^. The cell wall determines cell shape and protects cells against osmotic lysis^3^. It is assembled from peptidoglycan (PG), which consists of glycan chains with repeating disaccharide units of *N*-acetylglucosamine (GlcNAc) and *N*-acetylmuramic acid (MurNAc). Short peptides are attached to each MurNAc sugar and used to form amide crosslinks between adjacent glycans, generating a covalent mesh encapsulating the cytoplasmic membrane.

Rod-shaped cells such as *Escherichia coli* (*E. coli*) lengthen their cell body through the action of the elongation machinery (Rod complex, elongasome), which incorporates new PG material at dispersed locations throughout the cylinder^3^. Cell division is then initiated by the tubulin-like FtsZ protein, which at midcell forms the Z-ring that recruits dozens of proteins to the division site, assembling the divisome^4,5^. This apparatus promotes localized synthesis of PG to generate the cross-wall/septum that divides the daughter cell compartments^3^. The septal PG (sPG) produced initially connects the daughters such that it must be processed to separate the newly formed cells^3^.

Our understanding of cell envelope biogenesis during cell division has been greatly influenced by electron micrographs of developing septa^6^. However, sPG has not been clearly visualized in the septa of Gram-negative bacteria. Furthermore, whether the different septal architectures observed in diverse bacteria^7–12^ reflect fundamental differences in the division mechanism between species or arise from changes in the spatiotemporal regulation of conserved processes remains a major outstanding question. We therefore investigated the structure and dynamics of the septal PG layer of *E. coli* using both in situ cryo-electron tomography (cryo-ET) imaging and live-cell fluorescence microscopy.

## Results

### Architecture of the *E. coli* division site

**Fig. 1:**
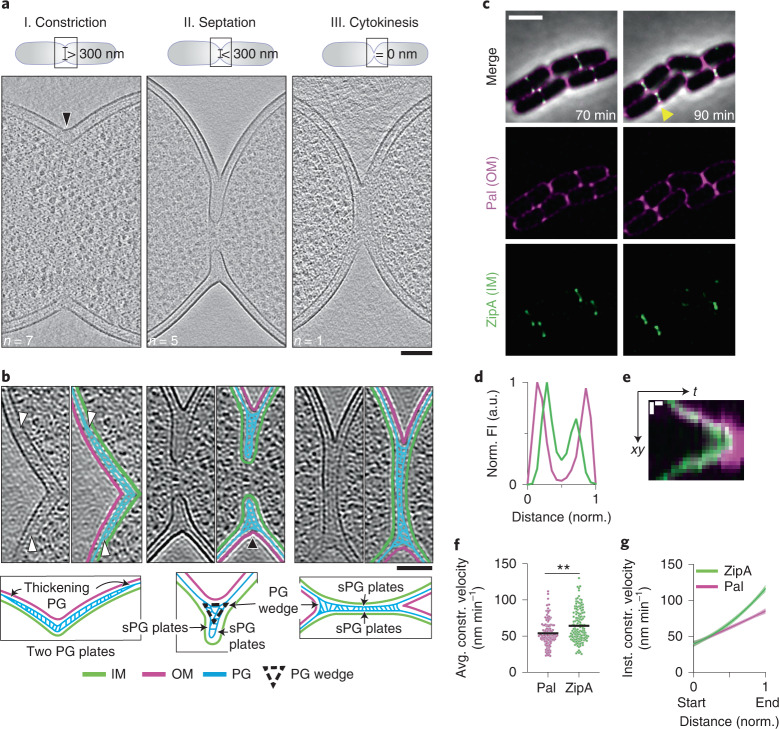
In situ cell envelope architecture and dynamics during *E. coli* cell division. **a**, Overview of different stages of cell division. Summed, projected central slices of cryo-electron tomograms visualizing different stages in division of wild-type *E. coli* are shown. Black arrowhead indicates the side of the division site displayed in (**b**). **b**, Top row: NAD-filtered cryo-electron tomograms visualizing the cell wall. Left panels show a 2D slice, right panels show the corresponding slice with segmentations for the observed PG signal in cyan, IM in green and OM in magenta (see Methods). White arrowheads indicate where the PG layer appears to thicken from one to two layers, and black arrowhead indicates the side of the division site shown in the schematic overview below. Bottom row: corresponding labelled summary diagrams. The left two bottom panels correspond to arrowhead-marked top division side rotated 90° to the left. Segmented PG signal is not indicative of specific glycan strand network. **c**–**e**, Representative time-lapse series from 3 biological replicates of wild-type *E. coli* expressing Pal-mCherry and ZipA-sfGFP as OM and IM markers, respectively, imaged at 30 °C on M9 supplemented with 0.2% casamino acids and d-glucose. Fluorescence signals were deconvolved (see Methods). The yellow triangle marks division sites used for line scans of fluorescence intensity (FI) profiles (**d**) and kymograph analysis of cytokinesis (**e**). **f**, Average constriction velocities of the IM and OM were derived from the slopes of the fluorescence signals in kymographs (see Methods). Black line indicates mean. Two-sided unpaired Mann-Whitney test; **P < 0.01; *N* = 150 division kymographs. **g**, Instantaneous constriction velocities for ZipA (IM, green) and Pal (OM, magenta) are plotted against normalized cell width. Second order polynomial fits with 95% confidence intervals are shown. Scale bars: **a** and **b**, 100 nm; **c**, 2 µm; **e**, 200 nm (vertical) and 5 min (horizontal). Source data

**Extended Data Fig. 1:**
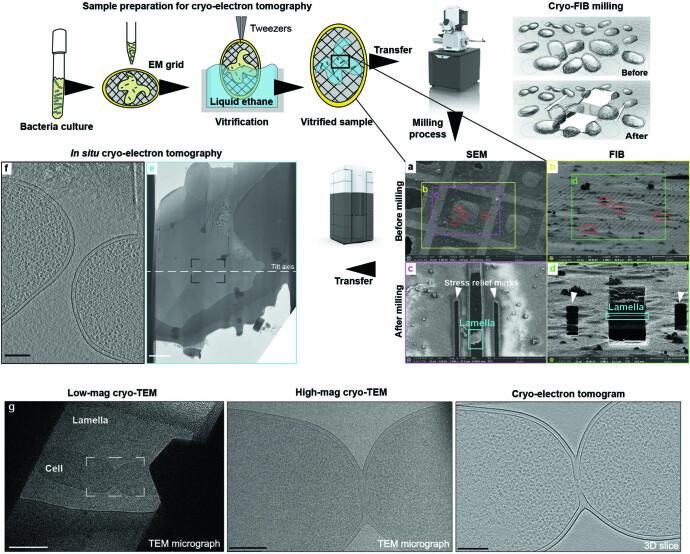
Cryo-FIB / cryo-ET pipeline utilized in this study. Schematic cartoons showing the steps in sample preparation for cryo-ET. In brief, bacteria are grown to OD_600_ = 0.3 and applied onto an EM grid for vitrification in liquid ethane^56^. Cryo-EM grids are kept in liquid nitrogen until transfer into the cryo-FIB microscope for milling. An illustration shows the result of milling vitrified bacteria distributed onto the holey carbon film on the mesh EM grid. (a-d) Images taken from the Aquilos Thermo Fisher Scientific graphical user interface during cryo-FIB milling performance. (**a**) Target bacteria (red circles) are first identified by SEM, *e*^*-*^ beam. Yellow box indicates region visualized in (**b**) and magenta box indicates the area visualized in (c). (b) Corresponding FIB, ion Ga^+^ beam, view (52° with respect to the *e*^*-*^ beam^58^) of the targeted grid square in (a) (yellow box). Green box indicates region visualized in (d). (**c**) SEM view of the same region shown in (a) and (b) after platinum deposition and milling. Cyan box indicates obtained lamella shown in (e). (**d**) FIB view of region shown in (b) (green box) after platinum deposition and milling. Scale bars in (a-d) are indicated on each image. After milling, cryo-EM grids containing bacterial lamellae are transferred into a TEM microscope. (**e**) Low magnification TEM 2D image of the lamella shown in (c-d), cyan box. Dashed black box indicates target region for cryo-ET acquisition. Dashed white line indicates the tilt axis for cryo-ET data acquisition. (**f**) 3D slice of the cryo-electron tomogram obtained from 3D reconstruction of aligned cryo-ET tilt series acquired in (e) (dashed black box). Scale bars: e = 1000 nm; f = 200 nm. (**g**) A representative lamella from a wild-type *E. coli* cell imaged at indicated imaging conditions. White box highlights region for corresponding high-magnification acquisition. Scale bars = 1 µm (low magnification); 200 nm (high magnification and cryo-electron tomogram).

**Extended Data Fig. 2:**
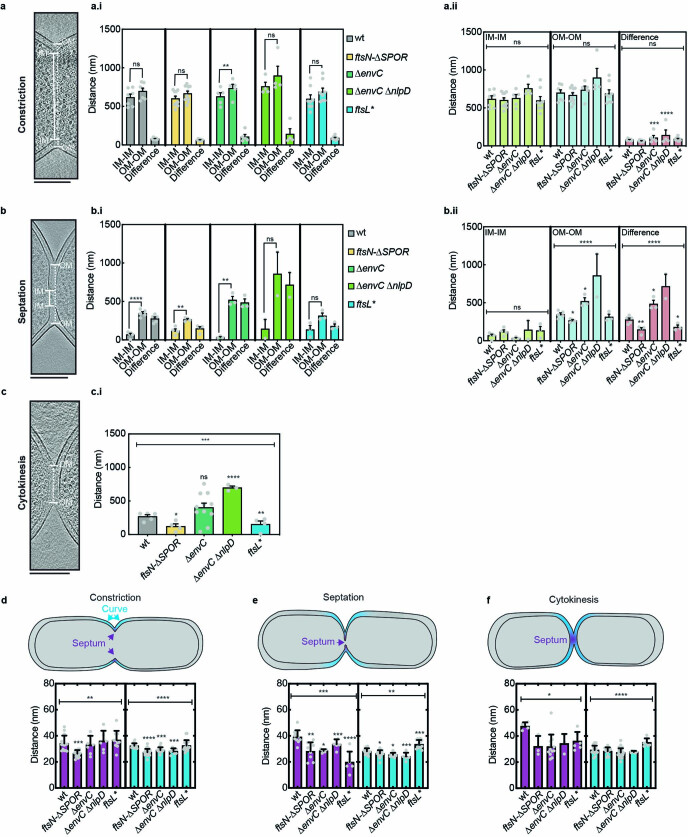
Distance measurements in cryo-ET data of dividing *E. coli* cells. Three dimensional slices visualizing the division site during (**a**) constriction, (**b**) septation and (**c**) cytokinesis. Dashed white line indicates OM-OM distance and white bold line indicates IM-IM distance. (a.i-c.i) Measured distances in nm of IM-IM, OM-OM and the difference between these distances at (a.i) constriction (N = 7 (wt); 10 (*ftsN-∆SPOR*); 5 (*∆envC*); 4 (*∆nlpD ∆envC*); 8 (ftsL*) images), (b.i) septation (N = 5 (wt); 4 (*ftsN-∆SPOR*); 3 (*∆envC*); 2 (*∆nlpD ∆envC*); 3 (ftsL*) images), and (c.i) cytokinesis (N = 5 (wt); 4 (*ftsN-∆SPOR*); 11 (*∆envC*); 3 (*∆nlpD ∆envC*); 4 (ftsL*) images). (a.ii-b.ii) Measured distances in nm for each strain grouped. Scale bars = 200 nm. All data are expressed as mean + SEM. (**d**-**f**) Schematic representing the division stages color-coded at where periplasmic width was measured. Thirty euclidean distances were measured per region (see Methods), N values for each region per stage are: (d) at septum (N = 14 (wt); 16 (*ftsN-∆SPOR*); 7 (*∆envC*); 6 (*∆nlpD ∆envC*); 10 (ftsL*)); at curve (N = 15 (wt); 27 (*ftsN-∆SPOR*); 13 (*∆envC*); 8 (*∆nlpD ∆envC*); 19 (ftsL*)); (e) at septum (N = 10 (wt); 8 (*ftsN-∆SPOR*); 6 (*∆envC*); 6 (*∆nlpD ∆envC*); 6 (ftsL*)); at curve (N = 19 (wt); 23 (*ftsN-∆SPOR*); 12 (*∆envC*); 6 (*∆nlpD ∆envC*); 12 (ftsL*)); (f) at septum (N = 4 (wt); 3 (*ftsN-∆SPOR*); 12 (*∆envC*); 3 (*∆nlpD ∆envC*); 6 (ftsL*)); at curve (N = 16 (wt); 12 (*ftsN-∆SPOR*); 39 (*∆envC*); 6 (*∆nlpD ∆envC*); 7 (ftsL*)). All analyzed data points are displayed, bar represents mean + SD. Significance was tested using unpaired t-test with Welch correction for paired groups when data followed gaussian distribution and Mann-Whitney when data did not follow gaussian distribution and tested relative to wild-type. Significance difference among all groups compared was tested using Welch ANOVA and Brown-Forsythe when data followed gaussian distribution and Kruskal-Wallis test when data did not follow gaussian distribution. In (a.ii) difference plot, statistical significance is shown based on F-test statistics. Ns = non-significant, * = p < 0.05, ** = p < 0.01, *** = p < 0.001, **** = p < 0.0001. Source data

**Extended Data Fig. 3:**
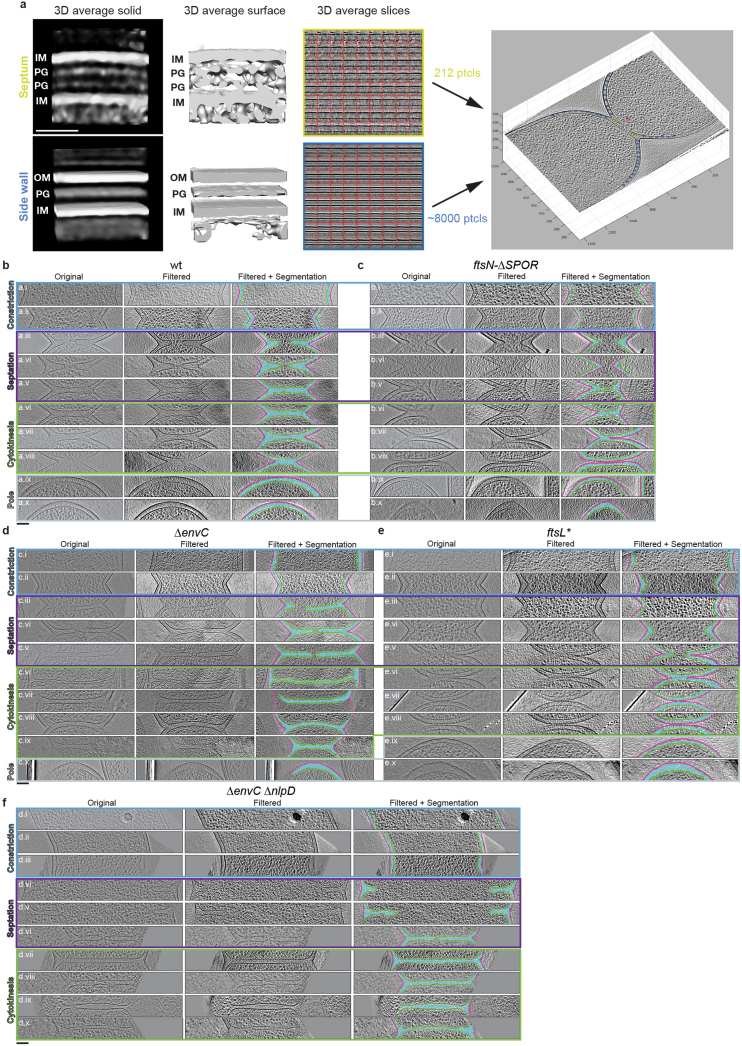
Subtomogram averaging, NAD filtering and segmentation of the cell envelope of *E. coli*. (**a**) STA 3D structure of the cell envelope at the septum and side wall are displayed in Chimera using solid and surface rendering. 3D slices of averages are shown. 212 particles contributed to the septum average while 8072 particles from N = 5 tomograms contributed to the side wall average. Blue dots plotted on a tomogram slice represent particles that contributes to ‘side wall’ and yellow dots represent particles contributing to septum average. Scale bar = 40 nm. In the tomogram rendering, 100 pixels blocks in the cartesian axes correspond to 102.6 nm. (**b**-**f**) Gallery of corresponding zoom-in summed projected central slices of cryo-electron tomograms visualizing the indicated division mutants. First column shows original image, second column shows filtered image, and third column shows filtered image with segmentation layers indicating IM = green, PG = cyan and OM = magenta. A full cryo-ET gallery can be found in Extended Data Fig. 5. A complete overview of the number of tomograms is reported in Supplementary Tables 2, 3. Scale bars = 100 nm.

Bacterial lamellae ~150–250 ﻿nm thick were generated by cryo-focused ion beam (cryo-FIB) milling^13–19^ for in situ cryo-ET imaging (Extended Data Fig. 1). A total of 22 tilt-series of wild-type cells were acquired and three-dimensionally (3D) reconstructed (Fig. 1a, and Supplementary Tables 1 and2). To gain better visualization of the sPG, nonlinear anisotropic diffusion (NAD) filtering was applied to denoise the cryo-electron tomograms (Fig. 1b and Supplementary Video 1). Densities corresponding to the OM, PG, and inner membrane (IM) were traced and 3D segmented (see Methods). Cells with an IM-IM distance >300 nm were classified as undergoing constriction. They had a V-shaped constriction with a relatively uniform invagination of the two membranes and an indented mesh of PG (Fig. 1a,b, Extended Data Fig. 2a and Supplementary Table 3). Cells classified as undergoing septation had an IM-IM distance <110 nm. They displayed a partial septum where the IM was more deeply invaginated than the OM, with an average difference of 138.5 ± 24.51 nm (Fig. 1a,b and Extended Data Fig. 2b). Strikingly, the denoised tomograms showed an elongated, triangular wedge of PG close to the invaginating IM (Fig. 1b). In cells at the final stages of cytokinesis, where IM fission was complete (Extended Data Fig. 2c), two layers of PG signal comprising the septum were readily visible (Fig. 1b). We performed subtomogram averaging to compare the envelope structure between the side wall and septum of dividing cells, which also showed two layers of PG signal within partial septa and a single layer of PG signal in the side wall (Extended Data Fig. 3a).

**Extended Data Fig. 4:**
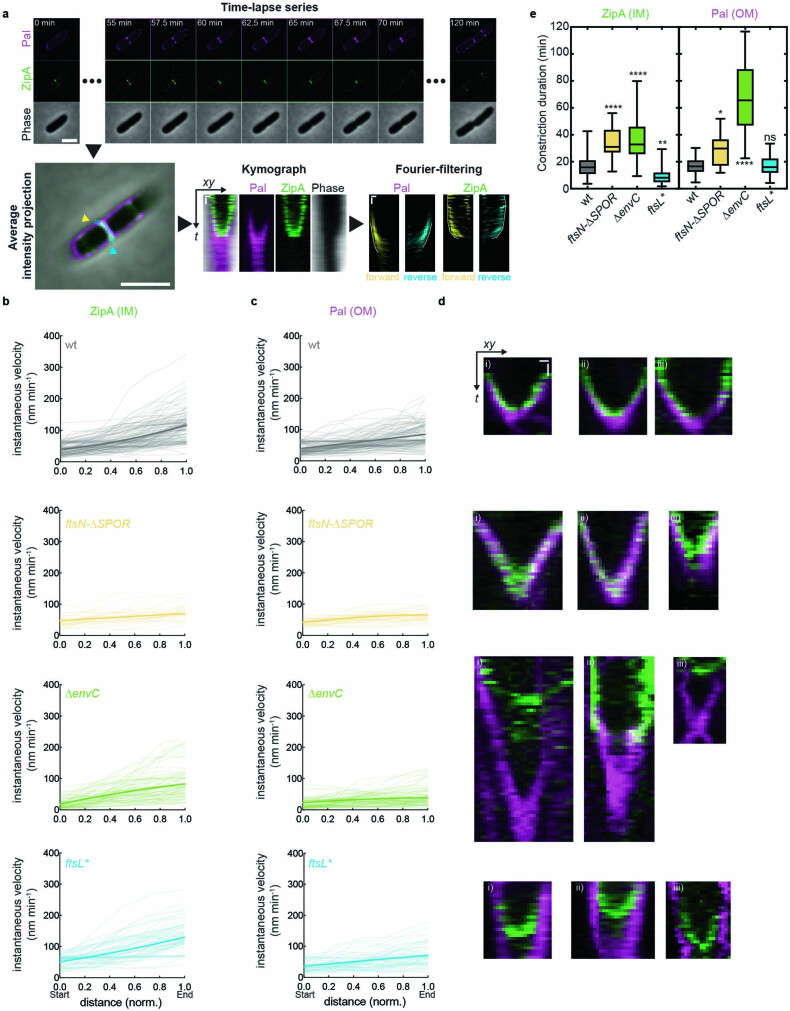
Measuring cell envelope constriction from kymograph data. (**a**) Schematic representation of workflow for the generation of kymographs using *Kymoclear* and *KymogrphaDirect* software. Instantaneous constriction velocity for (**b**) IM (ZipA-sfGFP) and (**c**) OM (Pal-mCherry) are plotted against normalized cell width. Bold lines show second order polynomial fits as in Fig. 2e,f. N = 150 cells (wt); N = 48 (*ftsN-∆SPOR*); N = 81 (*∆envC*)**;** N = 68 (ftsL*) kymographs. (**d**) Additional examples of cell envelope constriction kymographs for the corresponding strains in panels b-c. (**e**) Duration of IM (left) and OM (right) constriction was derived from kymograph measurements. Data are represented as boxplots. The line represents median; error bars depict Min-Max range. The significance of differences were tested relative to wild-type by one-way ANOVA with Dunnett’s correction for multiple comparisons; ns = non-significant (p = 0.99), * = p < 0.05, *** = p < 0.001, **** = p < 0.0001; N = 150 cells (wt); N = 48 (*ftsN-∆SPOR*); N = 81 (*∆envC*)**;** N = 68 (ftsL*). Scale bar = 2 µm, in kymographs = 200 nm horizontal and 10 min vertical. Source data

To investigate the mechanism of partial septum formation, we followed the constriction dynamics of each membrane. The IM was tracked using a superfolder green fluorescent protein (sfGFP) fusion to the IM-anchored Z-ring binding protein ZipA (ZipA-sfGFP), while constriction of the OM was followed using mCherry fused to the OM-lipoprotein Pal (Pal-mCherry) (Fig. 1c and Supplementary Video 2). The Pal-mCherry and ZipA-sfGFP signal distribution at the division site confirmed the deeper constriction of IM with respect to the OM (Fig. 1d) observed by cryo-ET (Fig. 1a,b). The invagination rate of each membrane was calculated from kymographs (Fig. 1e,f and Extended Data Fig. 4). We found that the IM constriction rate increased faster than linear as the septum closed, with an average rate of 64.26 ± 33.98 nm min^−1^, in line with previous measurements^20,21^. The increase in the OM constriction rate during division was less pronounced than that of the IM (Fig. 1f,g and Extended Data Fig. 4b–d). The different rates of change in constriction velocity between the two membranes account for the two membranes becoming increasingly separated as division proceeds, by 147 nm at late stages in cell division, which is in good agreement with our cryo-ET data. Thus, *E. coli* divides by a mixed constriction/septation mechanism, with the partial septum containing two layers of sPG signal (Fig. 1b and Extended Data Fig. 3a).

### sPG synthesis and remodelling defines septal architecture

**Fig. 2:**
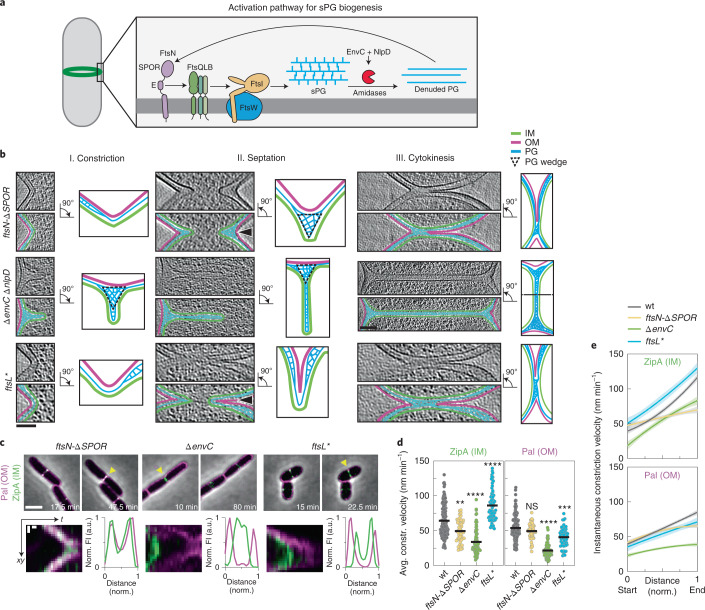
Divisome mutants display altered division site ultrastructure and constriction kinetics in *E. coli*. **a**, Schematic overview of the septal PG loop pathway for the activation of sPG synthesis (see text for details). **b**, Left: NAD-filtered cryo-electron tomograms of division sites in the indicated division mutants of *E. coli* shown as in Fig. 1b. Right: summary diagrams of the cell envelope architecture visualized. Black arrowheads indicate the side of the division site represented in the schemes. Segmented PG signal is not indicative of specific glycan strand network. **c**, Top: representative time-lapse series from 3 biological replicates of indicated *E. coli* division mutants expressing Pal-mCherry and ZipA-sfGFP as OM and IM markers, respectively, imaged as in Fig. 1. Bottom: kymograph analysis and line scans of fluorescence intensity profiles of cytokinesis, from division sites marked with yellow triangles in the top row. **d**, Constriction velocities of the IM and OM were determined as in Fig. 1. Black line indicates mean. Data from wild type are replotted from Fig. 1f for comparison. Brown-Forsythe and Welch ANOVA test with Dunnett’s correction for multiple comparisons, significance of differences is tested relative to wild type (wt); **P < 0.01, ***P < 0.001, ****P < 0.0001; NS, not significant (*P* = 0.09); *N* = 150 (wt), 48 (*ftsN-∆SPOR*), 74 *(∆envC*), 68 (ftsL*) kymographs. **e**, Instantaneous constriction velocities for IM (top) and OM (bottom) are plotted against normalized cell width. Second order polynomial fits with 95% confidence intervals are shown. See Extended Data Fig. 4b,c for individual instantaneous constriction velocity traces. Data from wild type are replotted from Fig. 1g for comparison. Scale bars: **b**, 100 nm; **c**, top row, 2 µm; bottom row kymographs, 200 nm (vertical), 5 min (horizontal). Source data

**Extended Data Fig. 5:**
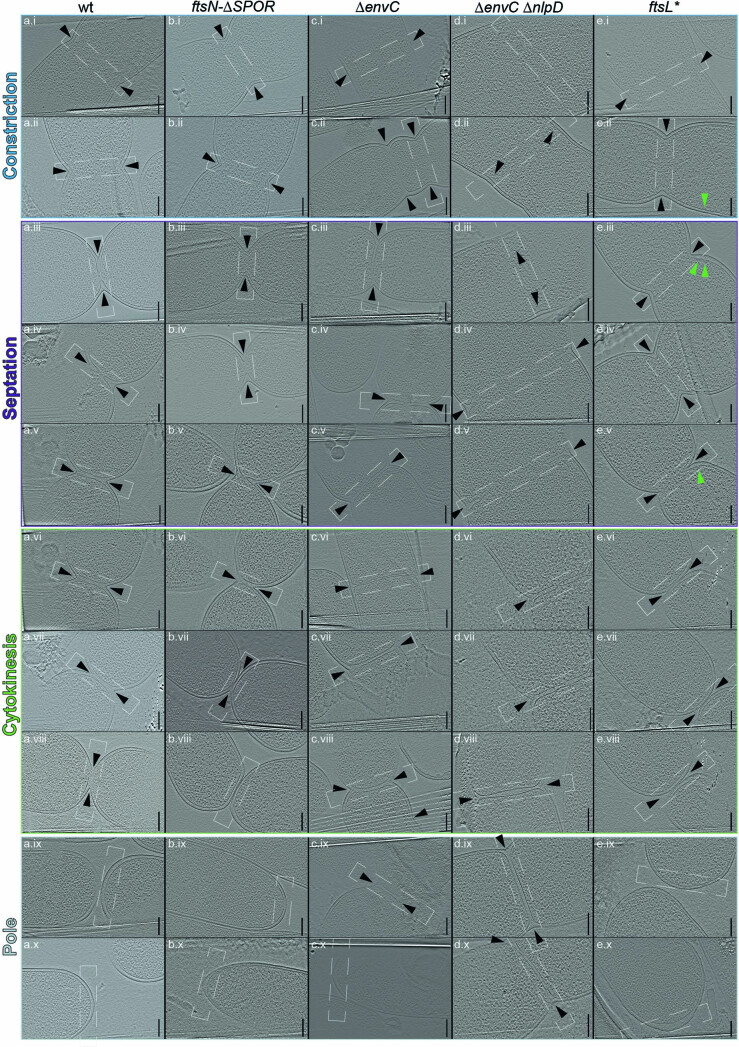
Cell division and polar morphology of *E. coli* viewed by cryo-ET. Gallery of summed projected central slices of cryo-electron tomograms visualizing the indicated division mutants. Black arrowhead = division site; green arrowhead = envelope bulging. Dashed white box indicates corresponding zoom-in region show in Extended Data Fig. 3. A complete overview of number of tomograms is reported in Supplementary Tables 2, 3. Scale bars = 200 nm.

**Extended Data Fig. 6:**
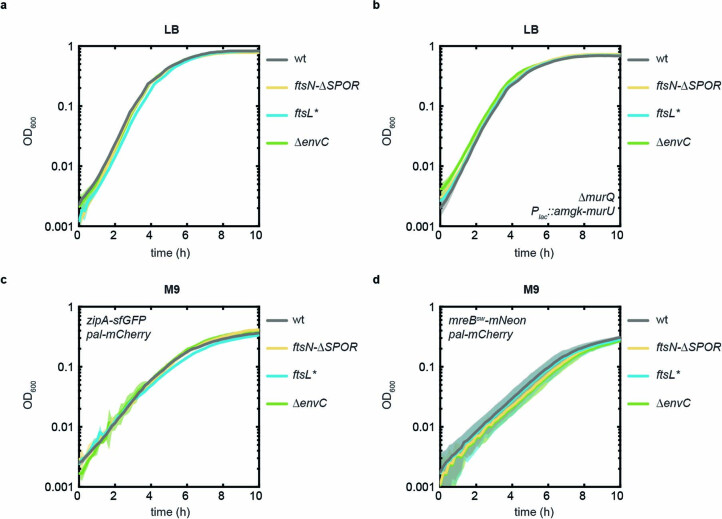
Measuring bulk growth rates of *E. coli* cell division mutants analyzed in this study. Growth curves were measured in biological triplicates by OD_600_ readings in a 96-well plate reader at 30 °C. Data is represented as mean ± SD. (**a**) Untagged strains used for cryo-ET and FDAA labeling as well as (**b**) *∆murQ* mutants expressing *amgK* and *murU* for MurNAc-alykyne labeling experiments were grown in LB. Cells harboring fluorescent fusion proteins for live-cell imaging of (**c**) cell envelope constriction or (**d**) MreB tracking were grown in M9 medium supplemented with 0.2% glucose and casamino acids. Source data

We next determined how the architecture of the division site and the dynamics of its constriction are altered by mutations affecting sPG synthesis and remodelling. The essential PG synthase of the divisome is formed by FtsW and FtsI (FtsWI)^22^. Following Z-ring assembly, a regulatory pathway is initiated that activates sPG synthesis by this synthase^23–26^(Fig. 2a). Activation is mediated in part via an interaction between FtsWI and the FtsQ-FtsL-FtsB (FtsQLB) complex^27^. Genetic evidence suggests that FtsQLB activation is stimulated by an essential peptide within FtsN^28^. Another domain of FtsN called SPOR concentrates the activation peptide at the division site through binding to sPG that was processed by PG amidases^28–30^. Amidases generate peptide-free (denuded) PG recognized by the SPOR domain as they split the sPG septum to promote OM constriction and daughter separation^31^. The interplay between sPG synthesis activation by FtsN and amidase processing bringing more FtsN to the division site promotes a positive feedback loop, the sPG loop, that has been proposed to drive cell division^28^. We imaged several mutants defective in this process (Supplementary Tables 4 and 5): (1) one lacking the SPOR domain of FtsN (*ftsN-∆SPOR*), (2) mutants defective for one (*∆envC*) or both (*∆envC ∆nlpD*) amidase activators^32^ and (3) a mutant (ftsL*) encoding a variant of FtsL that hyperactivates sPG synthesis^26^ (Fig. 2a, Extended Data Figs. 3b–f and 5, and Supplementary Tables 1–3 and 6). All mutants displayed similar growth rates (Extended Data Fig. 6).

Division sites from *ftsN-∆SPOR* cells resembled those observed previously for *Caulobacter crescentus*^33^ (Fig. 2b). By cryo-ET, there appeared to be greater coordination between IM and OM constriction throughout division (Fig. 2b, Extended Data Figs. 3b–f and 5, and Supplementary Video 3). Tracking of membrane constriction dynamics confirmed that the rate of constriction for the two membranes was nearly identical in the mutant (Fig. 2c–f, Extended Data Fig. 4b–e and Supplementary Video 2). As a result of the close opposition of IM and OM, the wedge of sPG observed in filtered tomograms was not as elongated as in wild-type cells, and separate plates of material forming ahead of the wedge were not observed (Fig. 2b).

Cells defective for both amidase activators (*∆envC ∆nlpD*) formed a near-complete septum in which the constriction of the IM was accomplished without much observable invagination of the OM (Fig. 2b, and Supplementary Figs. 3b–f and 4b–e**)**. NAD-filtering revealed signal corresponding to sPG that was even more clearly discernible as two distinct plates of material than in wild-type cells (Figs. 1b and 2b). Furthermore, the tomograms revealed a triangular wedge of PG material at the outer edges of the septa that was not previously observed in conventional EM analysis^34^ and presumably serves as a roadblock to OM invagination (Fig. 2b and Supplementary Video 4). It was not possible to measure membrane constriction dynamics in live cells of the *∆envC ∆nlpD* double mutant because it grew poorly when expressing the fluorescent markers. However, measurements in a mutant lacking only EnvC, the dominant amidase activator^32^, revealed a substantial disparity between IM and OM constriction rates (Fig. 2c–f and Supplementary Video 2). Cryo-ET data of the *∆envC* strain showed a similar division site architecture as the double *∆envC ∆nlpD* mutant (Extended Data Figs. 3b–f and 5). Overall, the results indicate that impairing key components of the divisome converts the mixed constriction/septation division mechanism of *E. coli* to either a purely constriction or septation mode depending on the lesion.

### Hyperactivated sPG biogenesis alters septal architecture

**Extended Data Fig. 7:**
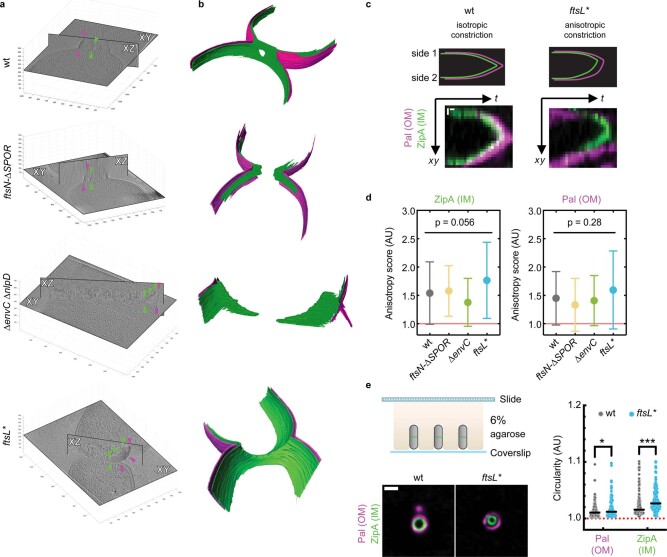
A hyperactivated divisome leads to anisotropic cell envelope constriction. (**a**) Orthogonal views of XZ and XY slices of 3D cryo-electron tomograms of the indicated division mutants. Magenta and green arrowheads indicate OM and IM, respectively. 3D volumes are displayed in cartesian 3D grids with axes indicating the dimensions in pixels. For wild-type and *∆envC ∆nlpD* 100 pixels = 102.6 nm, and for *ftsN-∆SPOR* and ftsL* 100 pixels = 110.3 nm. (**b**) Corresponding 3D surface segmentation renderings of OM (magenta) and IM (green) are shown on the right. (**c**) Schematic overview of a theoretical kymograph for an isotropic (left) and anisotropic (right) constriction of the cell envelope. Representative examples form 3 biological replicates for wild-type (left) and ftsL* (right) are provided. Scale bars: 200 nm (vertical); 5 min (horizontal). (**d**) An anisotropy score was calculated by taking the ratio of the constriction velocity from both sides of the cell. Red line (= 1) indicates a perfectly isotropic cell envelope constriction process. Data are represented as mean ± SD, Kruskal-Wallis with Dunn’s correction for multiple comparisons among all values was calculated, exact p values are shown. N = 65 (wt); 24 (ftsL*); 23 (*ftsN-∆SPOR*); 44 *(∆envC*) constriction were analyzed (**e**) Cells were vertically immobilized using small micro pillars imprinted into agarose pads, allowing to image the cell division site along its long axis. Representative example of the cell envelope position in vertically imaged wt and ftsL* cells. Scale bar = 2 µm. Circularity was quantified using *Morphometrics*. Red line (circularity = 1) indicates a perfect circle. Black line indicates median. Two-way ANOVA with Sidak’s multiple comparison test; * = p < 0.05; *** = p < 0.001. N = 132 (wt); 172 (ftsL*) cells imaged in three biological replicates. Source data

To determine the effects of hyperactivated sPG biogenesis on division site architecture, we imaged cells of the ftsL* mutant. Strikingly, cryo-ET revealed an altered architecture in which the signal corresponding to the wedge of sPG observed in wild-type and other mutant cells was missing, and the envelope at the leading edge of the invagination was 50.8% thinner than in wild-type cells (Fig. 2b and Extended Data Fig. 2d–f). Conversely, the envelope in the nascent polar regions adjacent to the leading edge of the invagination was 12% thicker in ftsL* cells than in wild-type cells, with the bulged areas containing more PG than normal (Extended Data Fig. 2e and Supplementary Video 5). As expected from previous measurements^26^, the average constriction velocity of the IM was much greater than in wild-type cells (Fig. 2c–e, Extended Data Fig. 4b–f and Supplementary Video 2). Surprisingly, the average rate of OM constriction was much slower than that of the IM (Fig. 2c–f and Extended Data Fig. 4), a difference that would normally be expected to give rise to cells with partial septa. However, such architectures were not observed in the tomograms (Fig. 2b, and Extended Data Figs. 3b–f and 5). The distances between the IM and OM remained relatively constant in all cells that were imaged (Fig. 2b, and Extended Data Figs. 2a–c, 3b–f and 5). This discrepancy is probably due to the PG binding activity of Pal causing the OM reporter to get stuck in the thicker PG that accumulates behind the closing septum and therefore to track poorly with the leading edge of the invaginating OM. Notably, upon closer inspection of kymographs of the ftsL* mutant, we noticed that one side of the cell constricted faster than the other (Fig. 2c). When we compared the constriction velocity for each side of the division site and assessed the degree of anisotropy (Extended Data Fig. 4a), ftsL* cells showed a higher but not statistically significant anisotropy score for both IM and OM constriction compared with other strains (Extended Data Fig. 7a–d). Additionally, when cells were imaged in vertical orientation, the constriction of ftsL* cells was less isotropic than in wild-type cells, indicative of uneven closure of the division ring (Extended Data Fig. 7e). Thus, circumvention of the normal controls regulating sPG biogenesis in the ftsL* mutant results in aberrant division site geometry and abnormal thickening of the envelope at the poles. These cells also lack an observable sPG wedge, which may destabilize the division site and help explain why these mutants were originally found to lyse at elevated temperatures^35,36^.

### sPG degradation activates its synthesis

**Fig. 3:**
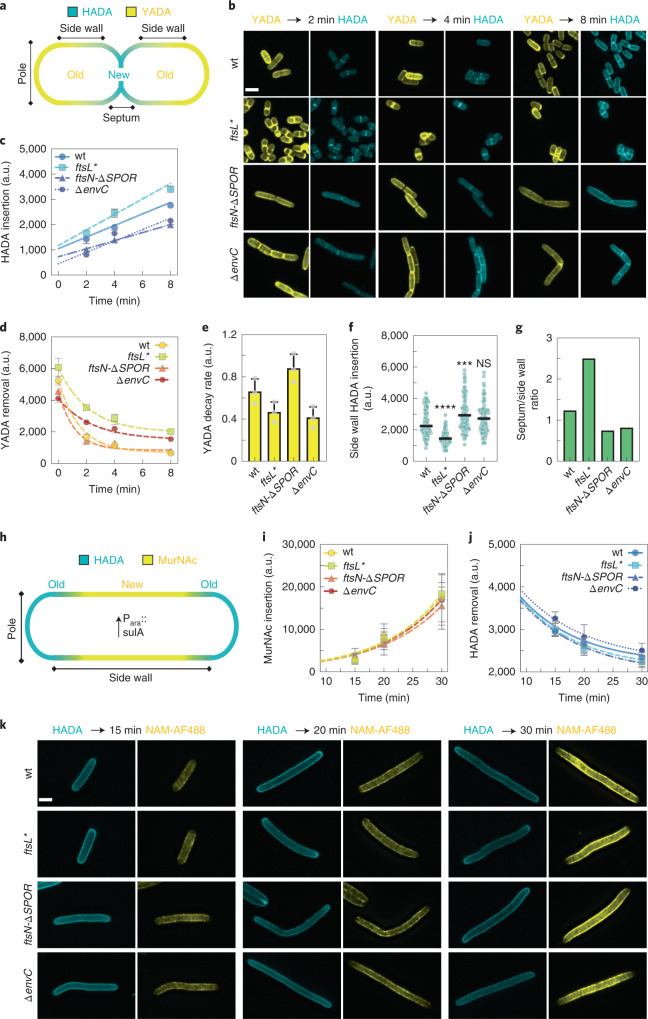
Measuring cell wall synthesis and hydrolysis rates during division and elongation in *E. coli*. **a**, Labelling patterns observed for an FDAA pulse-chase experiment. New cell wall material is labelled with HADA (blue), while old material is stained with YADA (yellow). **b**, Representative images from 3 biological replicates of indicated strains after 2, 4 and 8 min pulses with HADA. Overlay images are provided in Extended Data Fig. 8l,m. **c**,**d**, Mean fluorescence intensity was measured at the division site for new (**c**) and old (**d**) PG. **c**, Data were fit to a linear regression to derive sPG synthesis rates. Data points represent median ± 95% confidence intervals. **d**, Reduction in old (YADA) fluorescence intensity was fit to a one-phase exponential decay curve. **e**, Mean septal PG hydrolysis rates were derived from decay curves in **d**. Points represent the average value of the three biological replicates and bars indicate mean + 1 s.d. *N* = 1,054 (wt), 716 (*ftsN-∆SPOR*), 819 *(∆envC*), 880 (ftsL*) cells. **f**, Side wall incorporation of new cell wall material (HADA fluorescence intensity) was measured after 8 min due to low signal intensities in earlier time points. Black line indicates median, one-way ANOVA with Dunnett’s correction for multiple comparisons, significance of difference is tested relative to wild type; NS, *P* = 0.06; ***P < 0.001, ****P < 0.0001; *N* = 103 (wt), 107 (*ftsN-∆SPOR*), 101 *(∆envC*), 100 (ftsL*) cells. **g**, The ratio between sPG and side wall synthesis was calculated by dividing the mean HADA fluorescence intensity after the 8 min pulse. **h**–**j**, Labelling patterns observed for the pulse-chase experiment in cells with inhibited division by SulA expression (**h**). New cell wall material is labelled with Alexa488-labelled MurNAc-alkyne probes (yellow), while old material is stained with HADA (blue). Mean fluorescence intensity was measured along the side wall for both MurNAc-alkyne (**i**) and HADA (**j**) and fitted to a quadratic exponential Malthusian exponential growth function (**i**) or one-phase exponential decay (**j**). Data points represent median ± 95% confidence intervals. *N* = 578 (wt), 456 (*ftsN-∆SPOR*), 427 *(∆envC*), 501 (ftsL*) cells. **k**, Representative images from 3 biological replicates of indicated strains after 15, 20 and 30 min pulses with MurNAc-alkyne. Scale bar, 2 µm. Source data

**Extended Data Fig. 8:**
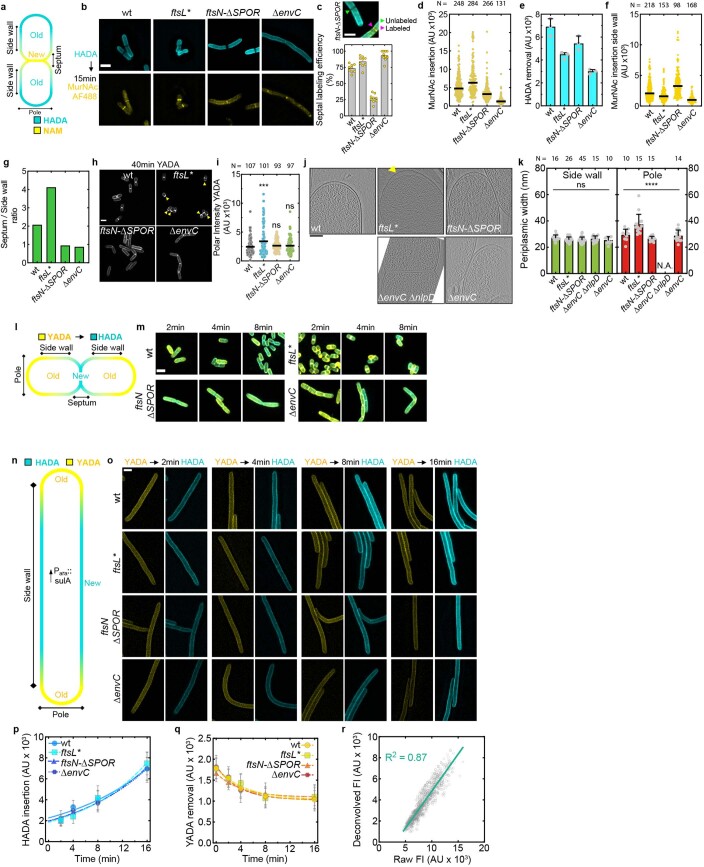
Cell wall synthesis and hydrolysis measurements. (**a**) New and old cell wall material were detected with Alexa488 labelled MurNAc-alykyne (yellow) or HADA (blue), respectively. (**b**) Representative images from labeling. (**c**) Label incorporation at the division site. Bars show the mean and dots show the average from 9 different images. *ftsN-∆SPOR* labeling example. (**d**) Rate of sPG synthesis (line = median). (**e**) Septal PG hydrolysis. Bar represents median + 95 % confidence interval; points indicate the average of three biological replicates. (**f**) Side wall labeling (line = median). (**g**) sPG and side wall synthesis ratio. (**h**) Representative YADA labeling pattern. Yellow arrow heads indicate polar label accumulation. (**i**) Average polar YADA fluorescence. Black line indicates mean, one-way ANOVA with Dunnett’s correction for multiple comparison. Significant differences are relative to wild-type; ns = non-significant (p = 0.76), p < 0.00. (**j**) Summed projected central 3D slices through tomograms of poles. Yellow arrowhead indicates enlarged periplasm. (**k**) Periplasm thickness in cryo-ET data at the side wall (green) and pole (red) (mean + SD). Thirty Euclidean distances were measured per region. One-way ANOVA (side wall) and Kruskal-Wallis test (pole); significance was tested among all groups within each region; ns = non-significant (p = 0.91), **** = p < 0.0001, N.A. = not applicable. (**l**) Labeling patterns observed for the pulse-chase. (**m**) Sum-projection of deconvolved images after HADA pulses as shown in Fig. 3b. (**n**) Expected labeling patterns for cells expressing *sulA*. New and old wall is labelled with HADA (blue) and YADA (yellow), respectively. (**o**) Representative images of indicated strains after HADA pulses. Mean side wall fluorescence intensity for (**p**) HADA and (**q**) YADA fit to a (**p**) Malthusian exponential function or (**q**) one phase exponential decay. Mean fluorescence intensity ± one SD is shown, N = 360 cells each. (**r**) Fluorescence intensity (**p**,**q**) was measured in original non-deconvolved (raw) SUM projections. Linear regression shows strong positive (R^2^ = 0.87) correlation to deconvolved fluorescence intensity values. N = 1035 values. Scale bars = 2 µm (fluorescence) or 200 nm (cryo-ET). Source data

To better understand the mechanism(s) by which changes in division site architecture are caused by mutations altering divisome components, we measured the rates of sPG synthesis and degradation using two different cytological assays (Fig. 3a and Extended Data Fig. 8a). The first assay used a pair of compatibly labelled fluorescent d-amino acids (FDAAs), YADA and HADA^37^, and the other used HADA and MurNAc-alkyne^20^. In both cases, cells were labelled extensively with an FDAA, pulsed with the second label for different lengths of time and then fixed before visualization. The intensity of the second label appearing at midcell after the pulse was used as a proxy for sPG synthesis. Additionally, the signal intensity of the first label before and after the pulse was used as a proxy for sPG degradation. Both assays yielded qualitatively similar results (Fig. 3a–g and Extended Data Fig. 8a–g). The ftsL* mutant synthesized sPG faster than all other strains just as it had the fastest rate of IM invagination (Figs. 2d and 3b,c, and Extended Data Fig. 8b–d). This result confirms that activated FtsQLB complexes indeed hyperactivate sPG synthesis, as suggested by recently reported effects on the dynamic motions of FtsWI^20^. Notably, the dual FDAA assay detected an increased amount of old PG at the division sites before the HADA pulse, and this material appeared to be relatively stable during the time course. Additionally, bright foci of old material were also observed at the poles of many cells after extended YADA labelling (Fig. 3b and Extended Data Fig. 8h,i). This accumulation of old material probably corresponds to the thickened areas of cell wall in nascent poles observed by cryo-ET of the ftsL* mutant (Extended Data Figs. 5 and 8j,k), reinforcing the conclusion that short-circuiting the normal controls governing sPG biogenesis not only leads to more rapid sPG synthesis and septal closure, but also aberrant accumulation of PG within the developing poles.

Both the *ftsN-∆SPOR* and *∆envC* mutants displayed a reduced rate of sPG synthesis relative to wild-type cells (Fig. 3b,c and Extended Data Fig. 8c,d). Although the sPG synthesis rates were similar, the two mutants differed in their rates of sPG degradation. The *ftsN-∆SPOR* mutant displayed relatively normal rates of sPG degradation, whereas *∆envC* cells showed reduced turnover of sPG as expected for a mutant lacking an amidase activator (Fig. 3d,e and Extended Data Fig. 8e). The combination of slower sPG synthesis with normal sPG degradation explains the well-coordinated constriction phenotype displayed by the *ftsN-∆SPOR* mutant in the cryo-ET analysis. The reduced rate of sPG synthesis in the *∆envC* cells is notable because it indicates that proper sPG processing by the amidases is required for normal rates of sPG synthesis. This result along with the reduced rate of sPG synthesis observed for the *ftsN-∆SPOR* mutant provides strong support for the sPG loop model^28^.

### sPG degradation is required for normal Z-ring formation

**Fig. 4:**
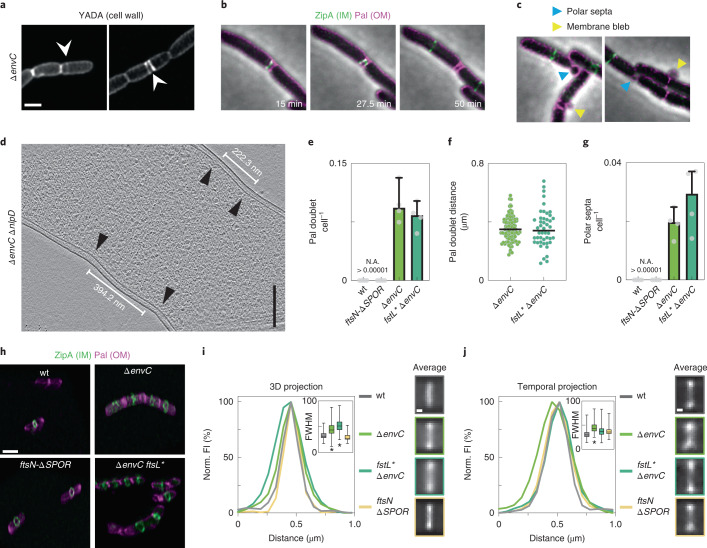
sPG hydrolysis is required for normal Z-ring placement and condensation in *E. coli*. **a**, Distribution of cell wall material in *∆envC* cells was assessed by FDAA staining in 3 biological replicates. Images are sum-projections of a 1 µm spanning *z*-stack and were deconvolved. White arrowheads indicate double septa. **b**, Representative time-lapse series from 3 biological replicates of a *∆envC* mutant expressing Pal-mCherry and ZipA-sfGFP as OM and IM markers, respectively. An example of double septum formation is shown. **c**, Examples of membrane blebbing (yellow arrowheads) and polar septa (blue arrowheads) formation are highlighted. **d**, Formation of double constrictions observed in cryo-electron tomograms of *∆envC ∆nlpD* cells. Black arrowheads indicate constriction sites. **e**, The frequency of double septum formation was quantified from counting the number of Pal-mCherry doublets per cell. No Pal doublets were found in >10,000 cells for wild-type or *ftsN-∆SPOR* cells in 3 biological replicates (N.A., not applicable). Data are represented as median + 95% confidence interval. **f**, The distance between Pal doublets was measured manually using the line tool in Fiji. *N* = 91 (*∆envC*), 46 (ftsL* ∆envC) Pal doublets measured. **g**, The frequency of polar septa per cell was measured for the indicated strains. No polar septa were observed in >10,000 wild-type or *ftsN-∆SPOR* cells. Data are represented as median + 95% confidence interval. **h**–**j**, Three-dimensional maximum intensity renderings showing Z-ring condensation based on ZipA-sfGFP localization (**h**). The degree of Z-ring condensation was quantified from averaged fluorescence intensity projections from summed 3D volumes (**i**) or from 5 time points (corresponding to 10 min) of a time-lapse series (**j**) (see Methods). Insets: FWHM of the fluorescence signal, with data represented as boxplots; line represents median, error bars depict minimum–maximum range. Inserts show average fluorescence intensity projection at the septum. Significance was tested against wild type by one-way ANOVA with Dunnett’s correction for multiple comparisons: *P < 0.05. *N* = 100 (wt, *∆envC*, ftsL* ∆envC, *ftsN-∆SPOR*) Z-rings from 3 biological replicates. Averaged Z-rings are shown and colour-coded according to graphs. Scale bars: **a**–**c**, 2 µm; **d**, 200 nm; **h**, 2 µm; **i** and **j**, 200 nm. Source data

Cells lacking EnvC commonly displayed closely spaced sPG labelling consistent with the aberrant formation of adjacent division sites (Fig. 4a). Accordingly, closer examination of the localization of Pal-mCherry in these cells revealed that double bands of the OM constriction marker occurred at an elevated frequency over wild-type or *ftsN-∆SPOR* cells (Fig. 4b,e,f). These double constrictions were also observed in cryo-ET (Fig. 4d and Extended Data Fig. 5) and were typically placed within the cell body. We also observed constrictions near cell poles, generating what appeared to be minicells (Fig. 4c,g). However, free minicells were not observed in the culture, suggesting that these aberrant poles were probably generated from a double constriction event within the cell body, one of which was aborted while the other completed division, generating a daughter with a polar constriction. Membrane blebs were also observed emanating from some of the developing septa of *∆envC* cells, some of which appeared to lyse, suggesting there was a catastrophic failure in division.

**Extended Data Fig. 9:**
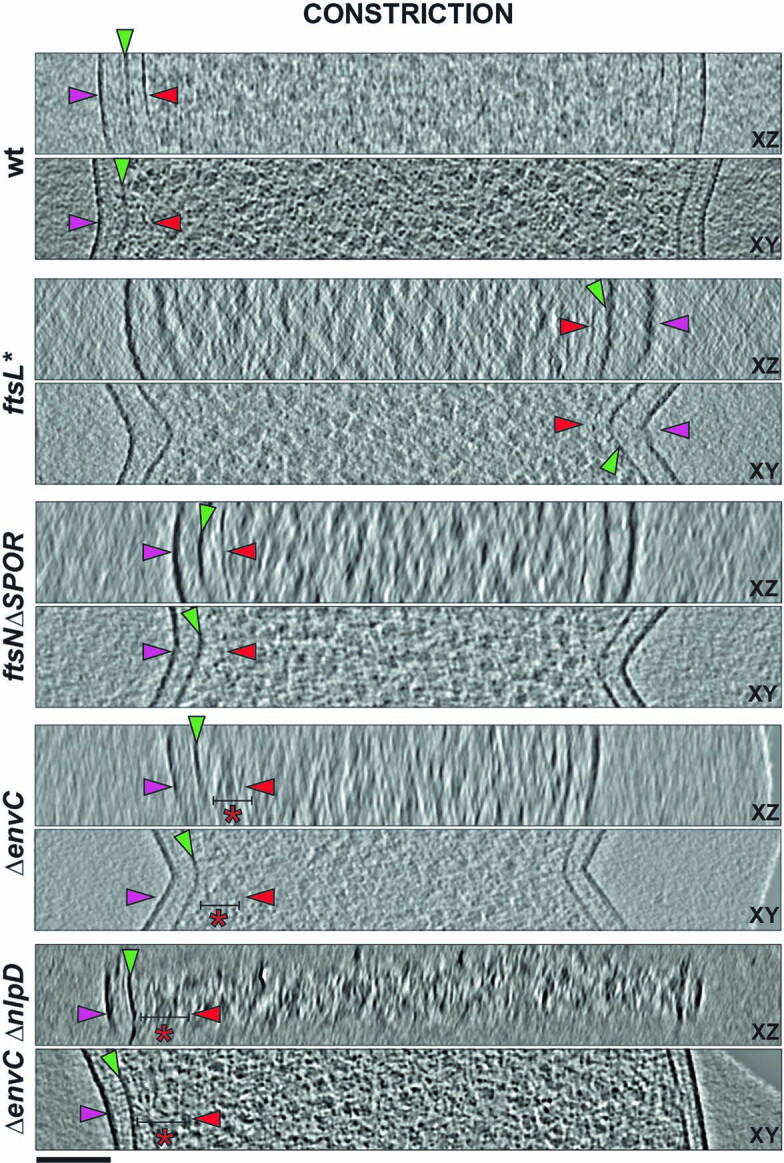
Z-ring views during constriction in cryo-electron tomograms of *E. coli*. Summed projections of 10 slices of XZ and XY views during constriction of indicated strains. Representative examples of all strains are shown. Green arrowheads indicate IM, magenta arrowheads indicate OM, red arrowheads indicate cytoskeletal ring and red asterisks indicate zones of diffuse signal. Note, Z-ring signal is weaker in *∆envC* mutants due to issues with Z-ring condensation as shown by fluorescence microscopy (Fig. 4h-j) Scale bar = 100 nm.

The pattern of ZipA-sfGFP was also altered in the *∆envC* mutant (Fig. 4h and Supplementary Video 6). ZipA binds FtsZ and is a Z-ring marker^38^. Many ZipA-sfGFP structures in *∆envC* cells were diffuse and/or malformed, indicating a difficulty condensing into the tight Z-ring structure typical of normal cells (Fig. 4h and Extended Data Fig. 9). Consistent with this possibility, averaging the ZipA-sfGFP signals for a population of cells, either over a 10 min time window or over a 2 µm volume spanning midcell, showed that the fluorescence was more broadly distributed in the *∆envC* mutant than in wild type or the other mutants (Fig. 4i,j). Notably, this phenotype was not suppressed by combining the ftsL* mutation with *∆envC*, indicating that it probably stems from the loss of sPG processing, not its collateral effect of reducing sPG synthesis (Fig. 4h–j). A diffuse Z-ring phenotype has been observed for cells defective in FtsZ-binding proteins such as ZapA that are thought to bundle FtsZ polymers to condense the ring^39,40^. These results therefore suggest a previously unappreciated role for sPG hydrolysis by the amidases in Z-ring condensation and division site stability and/or placement.

### Competition between elongation and sPG biogenesis

We took advantage of the PG labelling assays to quantify side wall PG synthesis (Fig. 3f and Extended Data Fig. 8f) and found that it was inversely correlated with sPG synthesis. Side wall PG incorporation was highest in the *ftsN-∆SPOR* mutant, which had one of the lowest rates of sPG synthesis (Fig. 3c,f,g and Extended Data Fig. 8d,f,g). Conversely, side wall PG synthesis was lowest in the ftsL* mutant that made sPG most rapidly (Fig. 3c,f,g and Extended Data Fig. 8d,f,g). In support of a competition with cell division being responsible for the differing rates of side wall PG synthesis, the rates were found to be the same in all cells when cell division was blocked (Fig. 3h–k and Extended Data Fig. 8n–q).

**Fig. 5:**
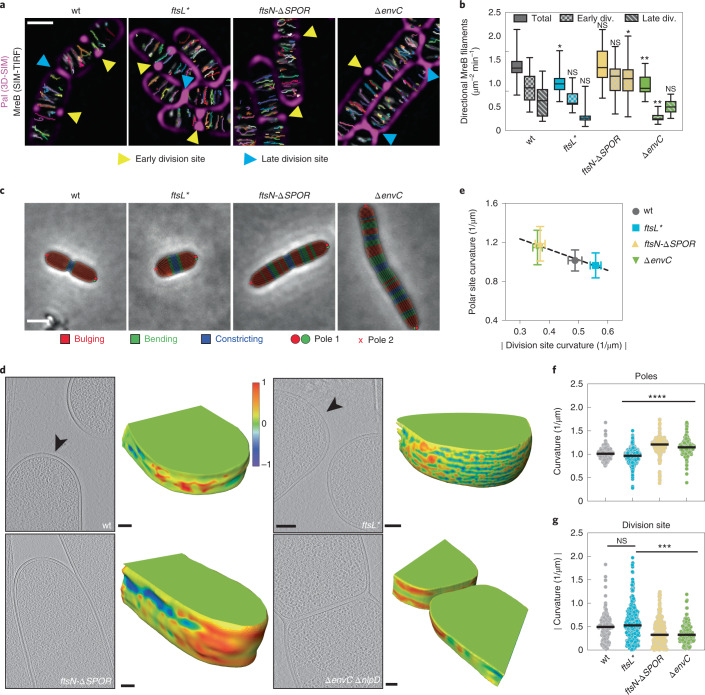
Competition between the divisome and elongation machinery defines polar cell shape in *E. coli*. **a**, MreB dynamics were followed by SIM-TIRF in indicated strains (see Methods). Time-lapse series were sum projected and overlayed with single-particle tracking results from TrackMate and 3D-SIM Pal-mCherry reference images. The Pal-mCherry signal serves to identify constricting cells. Early division site (yellow arrowheads) displayed Pal foci that were resolvable as two distinct foci, whereas late division sites (blue arrowheads) displayed a continuous Pal signal across the cell, indicative of complete or near-complete cytokinesis. **b**, Directionally moving MreB tracks were filtered by MSD analysis (see Methods), represented as boxplots (line indicates median; error bars depict minimum–maximum range) and normalized by cell area. Significance in each group was tested against wild type by one-way ANOVA with Dunnett’s correction for multiple comparisons: *P < 0.05, **P < 0.01; NS, *P* ≥ 0.05. *N* = 30 (wt, ftsN-∆SPOR, ∆envC, ftsL*) time-lapse series from 3 biological replicates. **c**, Representative phase-contrast micrographs showing segmented cells in ‘Morphometrics’ for the indicated division mutants. **d**, Summed, projected central 3D slices through cryo-electron tomograms of indicated strains visualizing cell poles. Black arrowheads indicate 3D-rendered pole. The corresponding 3D-volume renderings show polar curvature determined by shape index (see Methods). **e**–**g**, Polar curvature was measured by the two highest points of positive cell outline curvature (**f**), while constriction curvature was assessed by measuring the opposing contour-matched lowest curvature values at the division site (**g**) using Morphometrics and normalized to cell width (see Methods). Polar and division site curvatures are negatively correlated (*R*^2^ = 0.27) (**e**). Data are represented as mean ± s.d. For **f** and **g**, significance was tested against wild type by one-way ANOVA with Dunnett’s correction for multiple comparisons: ***P < 0.001, ****P < 0.0001; NS, *P* = 0.057. *N* = 460 (wt), 999 (ftsL*), 292 (*ftsN-∆SPOR*), 164 *(∆envC*) cells from 3 biological replicates. Scale bars: **a**, 1 µm; **c**, 2 µm; **d**, summed projection images, 200 nm and 3D renderings, 100 nm. Source data

**Extended Data Fig. 10:**
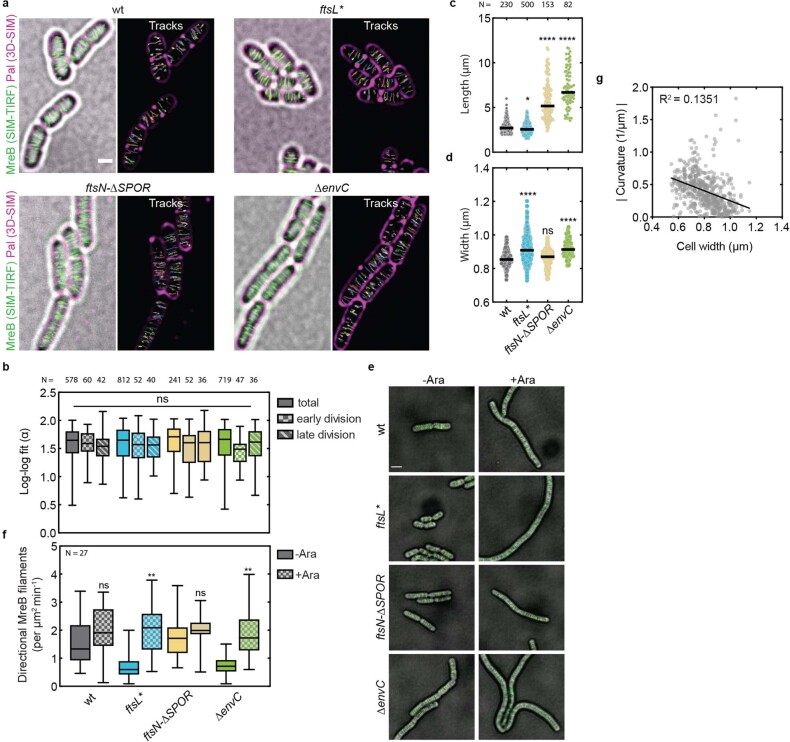
The balance between elongation and division affects cell morphology. (**a**) MreB-sw-mNeonGreen dynamics were followed by SIM-TRIF microscopy for 3 min at 3 s acquisitions per frame in indicated mutants. Time-lapse series was sum-projected and overlayed over a 3D-SIM Pal-mCherry and brightfield reference image. Larger fields of view are shown as compared to Fig. 5g and are representative from three biological replicates. Bar = 1 µm (**b**) Slopes of MSD curves (*α*) were analyzed following log-log fit to *l*og[*MSD*] versus *l*og[*t*] using the MATLAB class msdanalyzer^86^. Particles displaced by diffusive motion are characterized by a slope of their *l*og[*MSD*] = 1, while transported particles have slopes of 2 and constrained particles display slopes < 1. No significant difference (ns = non-significant, p = 0.0693; Kruskal Wallis test with Dunn’s correction for multiple comparisons) in the slopes of MSD curves were found indicating that MreB is displaced at a similar rate and manner in all strains. Box plot error bars displaying Min-Max range of values, blackline represents median. Tracks fit to *l*og[*MSD*] *l*og[*t*] with R^2^ ≤ 0.95 (**c**) Cell length and (**d**) width was measured from three independent biological replicates for the indicated mutants using *Morphometrics*. Line represents median. Differences in significances were tested relative to wild-type using Kruskal-Wallis one-way ANOVA with Dunnett’s correction for multiple comparisons; * = p < 0.05, **** = p < 0.0001, ns = non-significant, p = 0.69. (**e**) MreB dynamics were imaged as in (**a**). Representative temporal SUM projections of MreB-mNeon trajectories were overlayed to brightfield images. Bar = 2 µm. (**f**) Directionally moving MreB tracks were filtered by MSD analysis (see Methods) and represented as boxplots (line indicating median; error bars depict Min-Max range) and normalized by cell area. Significance in each group was tested against non-filamented control (-Ara) by two-sided unpaired t-test; ** p < 0.01, ns = non-significant, wt p = 0.053, *fstN-∆SPOR* = 0.253. (**g**) Constriction curvature values of wild-type cells are plotted against division site width in 566 cells. Linear regression (R^2^ = 0.135) indicates the negative correlation between cell width at the division site and constriction angle. Source data

Another measure of cell elongation activity is the circumferential motion of the Rod complex associated cytoskeletal element MreB around the cell cylinder^41–43^. We tracked the motion of an mNeonGreen fusion to MreB in wild-type and mutant cells using a combination of structured Illumination microscopy and total internal reflection fluorescence (SIM-TIRF) imaging. Consistent with the sPG synthesis measurements, the total number of directionally moving MreB filaments per area was significantly reduced in ftsL* cells (Fig. 5a,b, Extended Data Fig. 10a,b and Supplementary Video 7), which had an increased cell width (Extended Data Fig. 10c,d) indicative of reduced Rod complex activity^44^. All mutants displayed a similar density of directionally moving MreB filaments following the inhibition of cell division (Extended Data Fig. 10e,f and Supplementary Video 7), providing further support for a competition between the processes of elongation and division.

Notably, the interplay between cell elongation and division impacted the geometry of the division site and the shape of the daughter cell poles (Fig. 5c,d). The *ftsN-∆SPOR* mutant, which elongates more rapidly and constricts slower, displayed an elongated division site and a shallower OM invagination angle at midcell as compared with wild-type cells (Fig. 5e–g). This altered constriction geometry was also observable by cryo-ET and correspondingly gave rise to daughter cells with pointier poles than wild-type cells (Fig. 5c–g). On the other hand, the rapidly constricting ftsL* mutant formed daughter cells with relatively blunt cell poles (Fig. 5c–g).

We reasoned that the variation in division site and polar geometry among the different strains could be related to the activity of the Rod complex at or near the division site. The number of directionally moving MreB filaments in proximity (≤200 nm) to cell constrictions was therefore quantified (Fig. 5a,b and Supplementary Video 8). Such filaments were readily observed to pass through division sites in both early and late pre-divisional cells in all strains tested. Notably, however, the *ftsN-∆SPOR* mutant displayed more MreB tracks at the division site at late stages of division than all other strains, and the ftsL* mutant showed the least number of total MreB tracks at the division site (Fig. 5b). Thus, the density of MreB tracks at the division site for these cells correlates well with the steepness of the constriction site and the extent of cell pole elongation observed for the different strains. The outlier was the *∆envC* mutant, which had an inverted trend of having fewer directionally moving MreB tracks at early division stages than at later points (Fig. 5b). We suspect that this change is due to the defect in sPG splitting, which causes a steep curvature of the inner membrane at early points in division that is probably unfavourable for MreB localization^45,46^. However, at later stages when sPG processing eventually allows for slow constriction of the OM, this curvature probably becomes more favourable for MreB localization, allowing elongation to occur near the division site to generate a shallow constriction such as that of the *ftsN-∆SPOR* mutant. Overall, these results not only provide strong support for a competition between the PG biosynthetic machineries involved in cell elongation and division, but also highlight the potential for this competition to define the morphology of the daughter cell poles.

## Discussion

### Architecture of the sPG layer

**Fig. 6:**
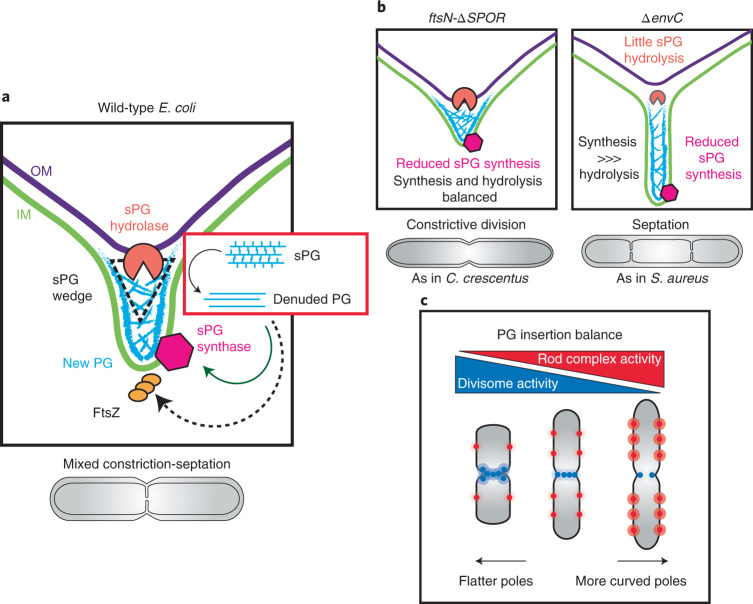
Septal PG architecture and divisome activity modulate bacterial morphogenesis in *E. coli*. **a**, Wild-type *E. coli* divides via a mixed constriction-septation mechanism in which a partial septum with two discernible plates of sPG is formed at later stages of the division process. A wedge structure is observable at the lagging edge of the septum where the dual layers of PG signal of the developing septum meet the single-layered signal of the side wall. Although not clearly resolved in the tomograms, we assume that the two layers of PG signal within the septum are probably connected by additional PG material (drawn as crosshatches). **b**, A constrictive mode of cell division is observed for the *ftsN-∆SPOR* mutant, where OM and IM invaginate at similar velocities due to lower sPG synthesis rates. The result is a V-shaped constriction that is similar to that formed by the distantly related Gram-negative bacterium *C. crescentus*. In contrast, inhibition of sPG hydrolysis causes a temporal separation of IM and OM constriction, leading to septation. These septa as well as the partial septa in wild-type cells are reminiscent of the Gram-positive bacterium *S. aureus*, which also displays two distinctive plates of sPG within its septa. **c**, The activities of the two major synthetic cell wall machineries, the Rod complex and the divisome, are anti-correlated probably due to competition for limited substrate (lipid II). The balance of their relative activities determines the shape of the cell division site and the resulting poles they form. Cells with higher Rod complex activity are thinner and form pointier poles, while cells with elevated divisome activity are shorter and wider, with blunt poles.

Here we combined cryo-FIB milling with cryo-ET to visualize the division site of *E. coli* in situ. In cells just starting to constrict, all three envelope layers appeared to be invaginating in concert, and little change in the sPG relative to the side wall PG was evident. However, the speed of IM invagination and sPG synthesis increases faster than PG splitting and OM constriction, leading to the formation of a partial septum (Fig. 6a) similar to that previously observed in fixed samples^9,10^. In NAD-filtered tomograms, a triangular wedge of what is likely to be sPG is observed at the lagging edge of the septum closest to the tip of the invaginating OM (Fig. 6a). The wedge thins as it approaches the leading edge of the closing IM. In this narrow portion of the septum, two dense tracks of material are often discernible, which correspond to the PG layers that will eventually fortify the daughter cell poles. In *ftsN-∆SPOR* cells with reduced sPG synthesis activity and slower IM constriction, a more uniform constriction of all envelope layers is observed, generating a division site architecture that resembles that of *C. crescentus*^33^ (Fig. 6b). However, in cells defective in sPG splitting, OM constriction is almost completely blocked and a Gram-positive-like septum is formed, with two visible tracks of PG reminiscent of the two tracks observed in the developing septa of *Staphylococcus aureus*^7^ (Fig. 6b). These results suggest that the activity of the same basic cell division machinery can generate different septal architectures observed in diverse bacteria. All that may be required is to change the relative activities of the sPG synthesis and remodelling systems.

In cells defective in sPG processing by the amidases, the sPG wedge structure is more prominent than in wild-type cells and it appears to impede the invagination of the OM. We thus infer that amidases process this structure to allow constriction of the OM (Fig. 6a). Furthermore, because the sPG wedge is observed in deeply constricted wild-type cells as well as unconstricted amidase activation mutants, we suspect that the structure is dynamic, with its lagging edge being degraded as new wedge material is deposited at the leading edge. Such a spatial separation of synthesis and degradation would allow the sPG wedge to move in a treadmill-like fashion ahead of the OM as the septum closes.

The enzymes responsible for creating the sPG wedge remain to be identified, but our results with the ftsL* mutant suggest that it is not made by FtsWI. This mutant is thought to hyperactivate FtsWI^20,24–27^. Therefore, if the wedge were produced by the FtsWI synthase, the ftsL* mutant would be expected to produce a thicker or otherwise larger wedge. Instead, it lacks a wedge altogether, suggesting that enhanced FtsWI activity disrupts biogenesis of the sPG wedge by other synthases. An attractive candidate for this additional synthase is the class A penicillin-binding protein (aPBP) PBP1b. Inactivation of PBP1b is synthetically lethal with defects in FtsWI activation. The affected mutants were found to lyse due to septal lesions, suggesting that this aPBP promotes division site stability^24,47^. The location of the wedge at the lagging edge of the division site closest to the OM is also consistent with a role for PBP1b in its construction, given that this enzyme requires an OM lipoprotein for activity^48,49^. Thus, the outer fork of the division site where the wedge is located is the only place where aPBPs would be predicted to be functional. Although further work will be required to test this model, it provides an attractive explanation for the division of labour between the aPBP and FtsWI synthases during constriction, with the FtsWI synthase promoting ingrowth of the PG layer and the aPBPs providing backfill to stabilize the septum and prevent lysis.

### The sPG activation loop

Our results support the proposal that FtsN and the amidases cooperate in a positive feedback loop that promotes sPG synthesis^28^. In addition to stimulating sPG synthesis, our results indicate that the sPG activation pathway also appears to be important for normal septal architecture. The ftsL* mutant hyperactivates the FtsWI synthase and eliminates the strict FtsN requirement for sPG biogenesis^26^. This short-circuiting of the normal division activation pathway not only causes the loss of the sPG wedge structure, but also promotes the aberrant accumulation of PG within the developing poles. Whether this accumulation results from inappropriate activation of PG synthesis by FtsWI or PBP1b, the improper turnover of the deposited material, or some combination of the two remains unknown. Nevertheless, what is clear is that bypassing the normal controls involved in sPG activation has adverse consequences on the architecture of the poles that are formed. We therefore infer that the normal divisome activation pathway serves an important function in coordinating different activities of the machinery to ensure that division is successfully completed once it is initiated and that the polar end products have a uniform surface.

### PG hydrolysis and the Z-ring

Our results have uncovered an unexpected connection between the activation of sPG processing by the amidases and the Z-ring structure, suggesting that there is feedback to the Z-ring from events downstream of sPG synthesis activation (Fig. 6a). Z-rings were found to be poorly condensed in mutant cells lacking the amidase activator EnvC (Fig. 4h–j**)**. Additionally, closely spaced constrictions or areas of sPG biogenesis were also observed at an elevated frequency in these cells, suggesting that division sites are unstable and fail before they complete the division process (Fig. 4a–g). Taken together, these results suggest the counterintuitive notion that sPG degradation by the amidases is required to stabilize the divisome, most probably via a positive influence on Z-ring condensation. Given that the amidases act on sPG in the periplasm, they are unlikely to directly modulate FtsZ activity. Rather, their effect is probably mediated through SPOR domain proteins such as FtsN and DedD that bind the amidase-processed glycans^29,30^. These proteins have transmembrane domains and N-terminal cytoplasmic tails, which in the case of FtsN is known to associate with the FtsZ-binding protein FtsA^50,51^. Thus, the status of sPG biogenesis in the periplasm could be communicated to the Z-ring in the cytoplasm using the binding of SPOR domain proteins to denuded glycans as a proxy. Whether the effect might be mediated simply by concentrating the cytoplasmic domains of SPOR proteins at the division site to modulate the activity of FtsZ-binding proteins or via more complex mechanisms requires further investigation, but the emerging picture is that the divisome activation pathway is not a one-way street from Z-ring formation to sPG synthesis and processing. The Z-ring probably also receives return stabilizing/activating signals from the PG biogenesis machinery.

### Cell shape and the balance between division and elongation

The idea that the cell elongation and division machineries may be in competition with one another has been discussed in the field for some time^52,53^. However, it has only been recently that evidence for such a completion has been presented^47,52,53^. Here we used several independent assays to demonstrate that septal and side wall PG synthesis rates are inversely correlated to each other, providing strong support for antagonism between the activities of the elongation and division systems, which most probably stems from a competition for the limited supply of the lipid II PG precursor. Importantly, our results indicate that this competition does not just affect cell width or how long or short cells are. It also influences the geometry of the septum and the shape of the daughter cell poles. Thus, modulation of the relative activities of the elongation and division systems is likely to play an important role in generating the diversity of shapes observed among different bacteria.

## Methods

### Media, bacterial strains and mutagenesis

Indicated strain derivatives of *E. coli* MG1655 used in this study are listed in Supplementary Tables 4 and 5. Bacteria were grown in LB (1% Tryptone, 0.5% yeast extract, 0.5% NaCl) or M9 media^54^ each supplemented with 0.2% d-glucose and casamino acids. For selection, antibiotics were used at 10 µg ml^−1^ (tetracycline), 25 µg ml^−1^ (chloramphenicol) and 50 µg ml^−1^ (kanamycin, ampicillin). Mutant alleles were moved between strains using phage P1 transduction. If necessary, the antibiotic cassette was removed using FLP recombinase expressed from pCP20^55^. All mutagenesis procedures were confirmed by PCR.

### Cryo-EM specimen preparation

Extended Data Fig. 1 summarizes the cryo-FIB/cryo-ET pipeline utilized in this study. Bacterial strains were grown overnight in LB media, back diluted 1:1,000 and incubated with shaking at 37 °C and 250 r.p.m. to optical density (OD)_600_ = 0.3. Cells were collected by centrifugation (2 min, 5,000 × *g*, r.t.) and resuspended in LB media to a final OD_600_ = 0.6. This cell suspension (3 µl) was applied to Cflat-2/1 200 mesh copper or gold grids (Electron Microscopy Sciences) that were glow discharged for 30 s at 15 mA. Grids were plunge-frozen in liquid ethane^56^ with an FEI Vitrobot Mark IV (Thermo Fisher Scientific) at r.t., 100% humidity with a waiting time of 10 s, one-side blotting time of 13 s and blotting force of 10. Customized parafilm sheets were used for one-side blotting. All subsequent grid handling and transfers were performed in liquid nitrogen. Grids were clipped onto cryo-FIB autogrids (Thermo Fisher Scientific).

### Cryo-FIB milling

Grids were loaded in an Aquilos 2 Cryo-FIB (Thermo Fisher Scientific). The specimen was sputter coated inside the cryo-FIB chamber with inorganic platinum, and an integrated gas injection system was used to deposit an organometallic platinum layer to protect the specimen surface and avoid uneven thinning of cells. Cryo-FIB milling was performed on the specimen using two rectangular patterns to mill top and bottom parts of cells, and two extra rectangular patterns were used to create micro-expansion joints to improve lamellae instability^57^. Cryo-FIB milling was performed at a nominal tilt angle of 14°−18°, which translates into a milling angle of 7°−11°^58^. Cryo-FIB milling was performed in several steps of decreasing ion beam currents ranging from 0.5 nA to 10 pA and decreasing thickness to obtain 100–200 nm lamellae.

### Cryo-ET

All imaging was done on an FEI Titan Krios (Thermo Fisher Scientific) transmission electron microscope operated at 300 KeV and equipped with a Gatan BioQuantum K3 energy filter (20 eV zero-loss filtering) and a Gatan K3 direct electron detector. Before data acquisition, a full K3 gain reference was acquired, and ZLP and BioQuantum energy filters were finely tuned. The nominal magnification for data collection was ×42,000 or ×33,000, giving a calibrated 4 K pixel size of 2.193 Å and 2.565/2.758 Å, respectively. Data collection was performed in the nanoprobe mode using the SerialEM^59^ or Thermo Scientific Tomography 5.3 software. The tilt range varied depending on the lamella, but was generally from −70° to 70° in 2° steps following the dose-symmetric tilt scheme^60^. Tilt images were acquired as 8 K × 11 K super-resolution movies of 4–8 frames with a set dose rate of 1.5–3 e^−^ Å^−1^ s^−1^. Tilt series were collected at a range of nominal defoci between −3.5 and −5.0 µm and a target total dose of 80–180 e^−^ Å^−2^ (Supplementary Table 1).

### Cryo-ET image processing

Acquired tilted super-resolution movies were motion corrected and Fourier cropped to 4 K × 5 K stacks, using ‘framealign’ from IMOD^61^. Tilt series were aligned using ‘etomo’ in IMOD^62^ and ‘Dynamo’. Contrast transfer function (CTF) estimation was performed in IMOD. CTF correction was performed using the ‘ctfphaseflip’ programme in IMOD^63^. CTF-corrected unbinned tomograms were reconstructed by weighted back projection with and without a SIRT-like filter and subsequently 2x, 4x and 8x binned in IMOD^62^.

Bandpass filtering and summed projection of cryo-tomogram slices were performed in Dynamo^64–67^ complemented with customized MATLAB scripts. Gaussian and NAD-filtering were performed in Amira (Thermo Fisher Scientific) for visualization purposes. NAD-filtering was applied using the command ‘Anisotropic Diffusion’ in 3D mode for 5 iterations. Gaussian filtering was done by applying the command ‘Gaussian Filter’ under 3D mode with a kernel size factor of 3. Whole 3D-volume FFT filtering was performed in IMOD.

#### Segmentation

Segmentation was performed on FFT filtered and NAD-filtered tomograms using Amira (Thermo Fisher Scientific) by non-biased semi-automatic approaches. Manual annotation was required every 10 slices, then Amira’s interpolation function was applied to automatically trace slices in between. Annotation was done in two-dimensional (2D) slices where features of interest were visible by eye. The segmented PG signal is not indicative of specific glycan strand network, but rather serves as a visual guide to relevant cell wall features.

#### Curvature

Three-dimensional pole curvature rendering was performed in Amira by applying the command ‘Curvature’ on the basis of the triangulated 3D mesh and ‘Shape Index’ as implemented in Amira^68^. Shape index (SI) computes the surface scalar field, which is calculated as

$$\mathrm{SI} = \frac{\pi }{2}\mathrm{atan}\frac{{C_1 + C_2}}{{C_1 + C_2}}$$

where *C*_1_ and *C*_2_ are the two principal curvatures. Shape index ranges from −1 to 1, negative values indicate negative curvature, positive values indicate positive curvature and values close to 0 indicate flatness of the surface. Values are normalized with respect to neighbouring triangles’ SI values^68^ (Fig. 5d).

### Quantification of cryo-ET data

#### Division site dimensions

Summed projection images of cryo-ET tomograms were used to quantitatively measure cell dimensions at the division site^69^. Measurements were performed in Fiji^70^ using the ‘point to point’ measuring tool. Measurements were from IM to IM and from OM to OM.

#### Periplasmic space

Measurements of periplasmic space thickness were performed from the centre of the OM to the centre of the IM in the cell areas referred to here as ‘side wall’, ‘pole’ and ‘curve’ as well as the invagination tip of the OM to the IM at the constriction division stage. Measurements from centre to centre of opposing IMs were performed in the cell area defined in this study as the septum (Supplementary Figs. 2 and 5**)**. We used a customized macro in Fiji that measures 30 Euclidean distances from surface-to-surface areas^71^ in nm, for example, from IM to IM at the septum and from IM to OM at the rest of the areas (side wall, pole, curve and initiation). For these 30 single measurements, the mean was calculated, yielding a final single value per defined subcellular localization, for example, septum, curve, pole and side wall.

### Subtomogram averaging

Subtomogram averaging was performed in Dynamo^64^. From the full wild-type cryo-ET data set, particles were identified using ‘dtmslice’ interface in Dynamo^66,67,72^. In 4x-binned tomograms, subtomograms with a size of (777.6)^3^ Å were extracted from 4x-binned tomograms. Initial angles were assigned following the normal of the IM. A starting reference generated from a random set of particles was used for both side wall and septum particles. A total of 16 iterations were used to align particles and obtain final averages. Final averages were generated from 8,076 subtomograms for the side wall and 212 particles for the septum. Notice that side wall regions were much more abundant in the cell than septum regions. EM densities were visualized in Chimera^73^.

### Sample preparation for live cell imaging

Overnight cultures of indicated *E. coli* strains were grown in LB supplemented with appropriate antibiotics at 37 °C. The next day, cells were collected by centrifugation (2 min, 5,000 × *g*, r.t.) and washed 2× with M9 medium. Day cultures were back diluted (1:1,000) and grown in M9 (0.2% d-glucose, 0.2% casamino acids) supplemented with 50 µM Isopropyl β-D-1-thiogalactopyranoside (IPTG) and appropriate antibiotics at 30 °C until OD_600_ = 0.2–0.4. For filamentation experiments, SulA was produced from pNP146^74^ by the addition of 0.2% l-arabinose during the last 10 min of the incubation period. Cells were collected (2 min, 5,000 × *g*, r.t.) and resuspended in 1/10th of the original volume. Two microlitres of this cell suspension were added onto a 1% (w/v) agarose in M9 (0.2% d-glucose, casamino acids) pad supplemented with 50 µM IPTG and covered with a #1.5 coverslip. For filamentation experiments, the agar pad was also supplemented with 0.2% l-arabinose.

### Live-cell imaging

All samples were imaged on a Nikon Ti-E inverted widefield microscope equipped with a fully motorized stage and perfect focus system. Images were acquired using a 1.45 NA Plan Apo ×100 Ph3 DM objective lens with Cargille Type 37 immersion oil. Fluorescence was excited using a Lumencore SpectraX LED light engine and filtered using ET-GFP (Chroma, 49002) and ET-mCherry (Chroma, 49008) filter sets. Images were recorded on an Andor Zyla 4.2 Plus sCMOS camera (65 nm pixel size) using Nikon Elements (v5.10) acquisition software. For subsequent deconvolution procedures, three 200 nm spaced *Z*-planes were acquired for both fluorescence channels using 100% LED output power and 50 ms exposure. Temperature was maintained at 30 °C using a custom-made environmental enclosure. After a 20 min acclimatization period, cells were imaged at a 2.5 min acquisition frame rate for a total observation time of 1–4 h.

### Image processing for fluorescence microscopy

First, time-lapse series and *Z*-stacks were drift corrected using a customized StackReg plugin in Fiji^70,75^. Subsequently, fluorescence images were deconvolved using the classical maximum likelihood estimation algorithm in Huygens Essential v19.10 (SVI), employing an experimentally derived point spread function (PSF) from 100 nm TetraSpeck beads (Thermo Fisher Scientific). Image reconstruction was performed over 50 iterations with a quality threshold of 0.01 and a signal-to-noise ratio set to 20 for live-cell imaging and 40 for fluorescent cell wall probes in fixed samples. Background removal was set to 0 to preserve fluorescence intensity values best among different images. Chromatic aberrations between different fluorescent wavelengths were post-corrected using the chromatic aberration corrector in Huygens from the TetraSpeck bead template. The same image reconstruction parameters and chromatic aberration templates were applied to images that were compared to each other. Last, reconstructed fluorescence images were merged back to phase-contrast images and rendered for figure or movie display with Fiji.

### Measuring cell envelope constriction dynamics

Fluorescent fusions to IM-anchored protein ZipA and OM-lipoprotein Pal allowed us to determine the respective positions of the different cell envelope layers during division. These cell envelope fiducial markers accumulate specifically during cytokinesis at the division site, which was critical for the generation of kymographs. Constriction dynamics of IM and OM were derived from kymographs generated using the Fiji plugin ‘KymographClear’^76^ and automatically split into forward and reverse trajectories using Fourier filtering. This filtering step allowed us to measure the constriction rate for each side independently. Constriction kinetics were derived by automatically extracting the fluorescent trajectories for ZipA and Pal using ‘KymographDirect’^76^ (Extended Data Fig. 4a). Anisotropy of the division process was determined by taking the ratio of the constriction velocities between the forward and reverse trajectories. Only cells where the division site displayed minimal signs of displacement except for constriction were analysed to eliminate confounding effects on the analysis by excessive cell movement (for example, pushing). This manual exclusion resulted in the rejection of approximately 15–20% of the cell division events. Applying these procedures, we found the constriction rate of the OM to be increasing over time, in contrast to a previously reported constant rate^77^. This might be explained by different image analysis procedures (for example, kymographs vs width measurements, Pal-mCh marker vs a combination of phase-contrast and FM4-64 dyes).

### Measuring division site circularity of vertically imaged cells

For vertical imaging of bacterial cells undergoing division, similar procedures as described previously^78,79^ were applied. A silicon wafer containing 5.5 µm long and 1.5 µm wide photo-resist pillars was generated following high aspect ratio photolithography procedures with an adhesion layer. The dimension of these pillars reaches the practically feasible aspect ratio for photolithography designs and thus impedes increasing pillar length without concomitantly increasing width, precluding use of elongated or chaining division mutants for this imaging mode. A modified silanization surface treatment with plasma cleaning was applied to increase the surface hydrophobicity of the silicon wafer to minimize agarose accumulation. Agarose micro holes were generated by pouring degassed 6% agarose (w/v) in H_2_O on the silicon wafer. Agarose was allowed to solidify for 40 min at r.t., was peeled off, cut into 5 ×5 mm pieces, and incubated in M9 medium supplemented with 0.2% d-glucose, casamino acids, 25 µg ml^−1^ chloramphenicol and 50 µg ml^−1^ ampicillin overnight.

Cells were grown as described for sample preparation for live-cell imaging and added on agarose pads. Cells that were not trapped in micro holes were washed off gently using 1 ml of growth medium. Five micrometre spanning *Z*-stacks (at a 200 nm step size) were acquired and subsequently deconvolved.

Circularity quantification was carried using the software package ‘Morphometrics’^80^. Fluorescence signals were segmented using Laplacian algorithm in combination with the peripheral fluorescence setting. Circularity (*C*) is calculated in Morphometrics as:

$$C = \frac{{P^2}}{{4{\Pi} \times A}}$$

where *P* is the perimeter and *A* is the area enclosed by the circle and is a dimensionless measure. A perfect circle displays a circularity of 1, while increasing values correspond to less circular objects. Cells that were trapped tilted in agar holes were manually excluded from the analysis (15 out of 573 analysed cells).

### Measuring Z-ring condensation from time-lapse data

Condensation of cytoskeletal elements was addressed using previously described procedures^40^. Briefly, five frames (corresponding to 10 min) from recorded time-lapse series were sum-projected in Fiji. Z-rings in these sum-projected images were then aligned along the length axis and average-intensity-projected into a single image. Fluorescence intensity was measured across the full width along the horizontal axis of the averaged projection image. Intensity values were normalized and their corresponding full width at half maximum (FWHM) values were calculated in MATLAB.

### Measuring Z-ring condensation from 3D data

Similar procedures as outlined for measuring Z-ring condensation in time-lapse series were applied. Two micrometre spanning *Z*-stacks (at a 200 nm step size) were acquired to capture a full 3D view of a cell. Images were restored in Huygens as described above. Image volumes were sum-projected into a single plane, Z-rings extracted, aligned and averaged as described above. Fluorescence intensity profiles were measured identically as for time-lapse data. Snapshots for 3D maximum intensity projections were rendered in Huygens.

### Measuring cell wall synthesis rates by biorthogonal MurNAc-alkyne probes

Septal cell wall synthesis rates were measured as described previously^81,82^. MurNAc-alkyne was purchased as a custom synthesis product from Tocris following the procedures of ref. ^81^. All experiments were carried out in *∆murQ* background and in the presence of pCF436^83^ for IPTG-inducible expression of AmgK and MurU. Filamentation was induced by expressing the FtsZ antagonist *sulA* from arabinose-inducible plasmid pNP146^74^. Overnight cultures were back diluted 1:1,000 into fresh LB containing 15 µg ml^−1^ gentamycin. Cells were grown at 37 °C until OD_600_ = 0.4. Subsequently, 1.5 ml of cells were collected (2 min, 5,000 × *g*, r.t.) and resuspended in 300 µl LB containing 1 mM IPTG and 0.5 mM HADA to label all cell wall material with FDAAs. For filamentation experiments, SulA expression was induced by the addition of 0.2% l-arabinose. Samples were incubated by rotating at 37 °C for 30 min. Endogenous UDP-MurNAc production was inhibited by the addition of 200 µg ml^−1^ fosfomycin. After 10 min incubation, cells were washed twice in 1.5 ml LB, 1 mM IPTG and 200 µg ml^−1^ fosfomycin. Next, cells were incubated for 15 min in the presence of 0.2% (w/v) MurNAc-alkyne, 1 mM IPTG and 200 µg ml^−1^ fosfomycin at 37 °C. Cells were fixed using ice-cold 70% (w/v) ethanol for 20 min at 4 °C. Next, cell pellets were washed 3× with 1x PBS. Biorthogonal MurNAc-alkyne probes were labelled by click chemistry using 5 µM Alexa488 azide substrate according to the manufacturer's instruction. Samples were stored in 20 µl PBS at 4 °C and imaged within 48 h of the labelling experiment.

Samples were imaged on a Nikon Ti2-E inverted widefield microscope equipped with a Lumencor Spectra III light engine, Semrock dichroics (LED-CFP/YFP/mCherry-3X-A-000, LED-DA/FI/TR/Cy5/Cy7-5X-A-000) and emission filters (FF01-432/36, FF01-515/30, FF01-544/24). Images were recorded using a 1.45 NA Plan Apo ×100 PH3 oil objective with Olympus Type F immersion oil and a pco.edge 4.2bi back illuminated cooled sCMOS camera using Nikon Elements 5.2.

One micrometre spanning *Z*-stacks (separated by 200 nm) were acquired and subsequently deconvolved as described above. *Z*-stacks were sum-projected using Fiji. De novo septal PG synthesis was assessed by measuring the mean fluorescence intensity of NAM-Alexa488 along the division site using the line tool (width, 3 pixels). Levels of cell wall hydrolysis were assessed by measuring the overall reduction in HADA fluorescence as compared to baseline signal intensity derived from fixing cells before MurNAc-alkyne chase. Reduction in fluorescence intensity of FDAAs is indicative of cell wall remodelling mediated by amidases, endopeptidases or transglycosylases.

### Measuring cell wall remodelling by FDAA incorporation

For FDAA pulse-chase experiments, cells grown overnight were back diluted 1:1,000 in fresh LB and grown until OD_600_ = 0.4 at 37 °C. For the filamentation experiment, *sulA* was expressed from pNP146^74^ by the addition of 0.2% l-arabinose to cultures during the last 10 min of the incubation period. Subsequently, 1.5 ml of cells were collected (2 min, 5,000 × *g*, r.t.) and resuspended in 300 µl LB containing 0.5 mM YADA. Samples were incubated while rotating at 37 °C for 40 min. Cells were washed once in 1.5 ml LB and resuspended in 300 µl LB containing 0.5 mM HADA. Samples were incubated at 37 °C for either 2 min, 4 min or 8 min before immediate fixation with 70% ethanol. After fixation, cells were washed 3× in PBS, stored in the dark at 4 °C and imaged within 48 h. The same image acquisition and analyses procedures were carried out as described for MurNAc-alkyne probes. Fluorescence intensity values were fit to a linear regression for HADA and an exponential one-phase decay for YADA. Levels of cell wall hydrolysis were assessed by subtracting the average fluorescence intensity from cells fixed before chase (0 min) and the respective time point, and fit to a linear regression model. Reduction in fluorescence intensity of FDAAs is indicative of cell wall remodelling mediated by amidases, endopeptidases or transglycosylases. In addition to the division site, fluorescence intensity measurements were also performed along the side wall and polar region of the cells at the 8 min time point. For filamenting cells, HADA fluorescence intensity values were fit to a Malthusian exponential equation, assuming cells keep elongation at the same rate before SulA induction.

### Bulk growth curve measurements

Overnight cultures of indicated *E. coli* strains were grown in LB supplemented with appropriate antibiotics at 37 °C. The next day, cells were collected by centrifugation (2 min, 5,000 × *g*, r.t.) and washed 2× with the respective growth medium (M9 or LB). Day cultures were back diluted (1:1,000) and grown in the respective media supplemented with corresponding IPTG concentration (50 µM for ZipA-sfGFP induction, 1 mM for AmgK/MurU expression) and appropriate antibiotics at 30 °C until OD_600_ = 0.3. Cells were collected (2 min, 5,000 × *g*, r.t.) and resuspended to an initial OD_600_ of 0.01 in a final volume of 100 µl. Growth curves were measured in a Tecan M-plex 96-well plate reader by OD_600_ read out. Plates were incubated with shaking at 30 °C for a total of 18 h.

### Cell shape quantification analyses

Bacterial cells were segmented and analysed from phase-contrast images using the software package ‘Morphometrics’^80^. Results from Morphometrics were post-processed using customized MATLAB scripts to exclude erroneously segmented cell debris in live-image data on the basis of area. Cell width, length and pole curvature per segmented cell were directly extracted from Morphometrics. Since curvature ($k = \frac{1}{r}$, where *r* is the radius of the cell cylinder) is dependent on the cell cylinder width, curvature values were normalized by multiplying half-cell width to each respective curvature value. Thus, spherical poles display curvature values of *k* = 1, while pointy (elongated) poles display elevated curvature values (*k* > 1) and flat (shortened) poles display reduced curvature values *k* < 1, respectively. We obtained division site curvature from both sides of the cell at the invagination site. The invagination site is defined as the narrowest segment of the cell, for example, lowest cell width value, that presents negative curvature on both sides of the cell body. Division site curvature was normalized to the half-cell width of the invagination site.

### SIM-TIRF microscopy and MreB tracking

Samples were prepared as described for live-cell imaging. To block cell division, *sulA* was expressed from pNP146^74^ by the addition of 0.2% l-arabinose during the last 10 min of the incubation period. Cells were added to high precision #1.5 coverslips (Marienfeld) and placed on a 1% (w/v) agarose pad in M9 (0.2% d-glucose, casamino acids, supplemented with 0.2% l-arabinose for filamentation experiments) and imaged at room temperature on a Nikon Ti2 N-SIM microscope equipped with N-SIM spatial light modulator illuminator, TIRF Lun-F laser combiner with 488 and 561 nm laser lines, an N-SIM 488/561 dual band dichroic mirror, SR HP Apo TIRF ×100 1.5 NA oil objective with automated correction collar and a Hamamatsu Orca Flash 4.0 camera attached to a Cairn Research Twimcam splitter with an ET525/50m or an ET605/70m emission filter (for MreB-sw-mNeonGreen or Pal-mCherry fusion, respectively). The refractive index of the immersion oil (1.512) (GE Healthcare) was optimized for MreB-sw-mNeonGreen signal and corrected using the automated correction collar for the Pal-mCherry fusion. Alignment of the 488 and 561 lasers for SIM-TIRF and 3D-SIM, and of the N-SIM optics and illumination was performed before each experiment at the image plane. First, a 3 min time-lapse series (at 3 s acquisition frame rate) in SIM-TIRF mode was collected using 20% laser power with 100 ms exposure time to follow MreB-sw-mNeonGreen dynamics. Then, a single slice of a 3D-SIM Pal-mCherry (40% laser power, 100 ms exposure) and a brightfield reference image was acquired. Raw fluorescence images were reconstructed using Nikon Elements 5.11 acquisition software with indicated settings: MreB illumination contrast 0.8, noise suppression 0.3 and blur suppression 0.05; Pal illumination contrast 3.75, noise suppression 0.1 and blur suppression 0.5. Only reconstructed images with a quality score ≥8 and passed SIMcheck quality test^84^ were used for further analysis. Subsequently, MreB time-lapse series were overlayed over the reference channels in Fiji.

Particle tracking was performed in Fiji using the TrackMate v6.0.1 plugin^85^. MreB filaments were detected using the LoG-detector with an estimated radius of 0.3 µm. Spurious spots were filtered using a quality threshold of 50. Spots were linked using a Kalman filter with an initial search radius of 0.2 µm and search radius of 0.1 µm. No frame gaps were allowed. Only tracks consisting of ≥4 continuous spots (12 s) and that travelled less than 1 µm in total distance were kept for further analysis. To analyse the nature of the displacement of each track, the mean square displacement (MSD) was calculated using the MATLAB class msdanalyzer^86^. Slopes (*α*) of the individual MSD curves were extracted using the log-log fit of the MSD and the delay time *τ*. As the maximum delay time of 75% of the track length was used, tracks with an $R^2\,{{{\mathrm{for}}}}\,{{{\mathrm{log}}}}\left[ {\mathrm{MSD}} \right]$ versus log[*t*] below 0.95 indicative of a poor fit to the MSD curve were excluded from the analysis. MreB filaments engaged in active cell wall synthesis are displaced by the enzymatic activities of RodA and PBP2b^41–43^, hence their MSD curves display slopes of *α* ≈ 2 indicative of a transported particle motion above the rate of Brownian diffusion (Extended Data Fig. 10b). MreB filaments in constricting cells, as determined by the presence of Pal-mCherry foci at the division site, were analysed by fitting a 200 nm wide region of interest to the cell division site. Directional MreB tracks were deemed to contribute to the elongation of the division site. Early and late division stages were distinguished by the presence of two separated Pal foci or a continuous fluorescent signal across the cell, respectively.

### Statistical analysis

All data measurements were plotted and analysed using GraphPad Prism 9 (Version 9.3.1). In general, (log-) normal distribution was tested using Shapiro-Wilk test. For comparisons of two groups, significance was determined by two-tailed, unpaired Student’s *t*-test with Welch correction and *F*-test for variance analysis. One-way analysis of variance (ANOVA) was used for comparison of more than two groups using the recommended post-test for selected pairwise comparisons. All experiments were carried out with at least 3 independent biological replicates. *P* values less than 0.05 were considered statistically significant.

### Reporting summary

Further information on research design is available in the Nature Research Reporting Summary linked to this article.

## Supplementary information

Supplementary Information Supplementary Figs. 1–10, Tables 1–6 and Video legends 1–8.

Reporting Summary

Supplementary Video 1 Supplementary Video 1. In situ cell division of wild-type *E. coli*. Cryo-electron tomograms of wt *E. coli*. Time-lapse series were acquired at a rate of 7 fps in the compressed format m4v for visualization purposes. Green, cyan and magenta layers indicate segmented IM, PG and OM, respectively. Scale bars, 100 nm.

Supplementary Video 2 Supplementary Video 2. Fluorescence live-cell imaging of cell envelope constriction in *E. coli* division mutants. Three time-lapse series of each indicated *E. coli* mutants acquired at a 2:30 min:sec acquisition interval are shown. Bacteria were imaged at 30 °C on 1% agarose in M9 supplemented with 0.2% casamino acids and d-glucose. Fluorescence channels (Pal-mCherry, magenta; ZipA-sfGFP, green) were deconvolved. The video was rendered at 12 fps. Scale bar, 2 µm.

Supplementary Video 3 Supplementary Video 3. In situ cell division of ftsN-∆SPOR. Cryo-electron tomograms of ftsN-∆SPOR mutant. Time-lapse series were acquired at a rate of 7 fps in the compressed format m4v for visualization purposes. Green, cyan and magenta layers indicate segmented IM, PG and OM, respectively. Scale bars, 100 nm.

Supplementary Video 4 Supplementary Video 4. In situ cell division of *∆envC ∆nlpD*. Cryo-electron tomograms of *∆envC ∆nlpD* mutant. Time-lapse series were acquired at a rate of 7 fps in the compressed format m4v for visualization purposes. Green, cyan and magenta layers indicate segmented IM, PG and OM, respectively. Scale bars, 100 nm.

Supplementary Video 5 Supplementary Video 5. In situ cell division of ftsL*. Cryo-electron tomograms of ftsL* mutant. Time-lapse series were acquired at a rate of 7 fps in the compressed format m4v for visualization purposes. Green, cyan and magenta layers indicate segmented IM, PG and OM, respectively. Scale bars, 100 nm.

Supplementary Video 6 Supplementary Video 6. Cell wall hydrolysis contributes to *Z*-ring condensation. Three-dimensional maximum intensity projections rendered in Huygens (SVI) of indicated *E. coli* strain expressing Pal-mCherry (magenta) and ZipA-sfGFP (green) are shown. The video was rendered at 12 fps. Scale bar, 2 µm.

Supplementary Video 7 Supplementary Video 7. MreB filament increase in cells with blocked cell division. Cell division was inhibited by expressing SulA using 0.2% l-arabinose. A three-minute SIM-TIRF time-lapse series of MreB-sw-mNeonGreen (green) was overlayed over a bright field reference image. The video was rendered at 12 fps. Scale bar, 2 µm.

Supplementary Video 8 Supplementary Video 8. MreB filaments regularly pass either through or in direct proximity of the cell division site. A three-minute SIM-TIRF time-lapse series of MreB-sw-mNeonGreen (green) was overlayed over a 3D-SIM image of Pal-mCherry (magenta) and bright field reference image. On the right side, tracking results from TrackMate are overlayed. The video was rendered at 12 fps. Scale bar, 1 µm.

## Data Availability

The data, plasmids and strains that support the findings of this study are available from the corresponding authors on reasonable request. Representative tomograms are deposited in EMDB: EMD-27479 (wild-type), EMD-27484 (*ftsN-∆SPOR*), EMD-27485*(∆envC* and *∆envC ∆nlpD*) and EMD-27486 (*ftsL**). Corresponding raw movie frames and stacks of tilt-series are deposited as EMPIAR-11090 (wild-type), EMPIAR-11087 (*ftsN-∆SPOR*), EMPIAR-11089 (*∆envC* and *∆envC ∆nlpD*) and EMPIAR-11088 (*ftsL**). Source data are provided with this paper.

## Source data

Source Data Fig. 1 Numerical data.
Source Data Fig. 2 Numerical data.
Source Data Fig. 3 Numerical data.
Source Data Fig. 4 Numerical data.
Source Data Fig. 5 Numerical data.
Source Data Extended Data Fig. 2 Numerical data.
Source Data Extended Data Fig. 4 Numerical data.
Source Data Extended Data Fig. 6 Numerical data.
Source Data Extended Data Fig. 7 Numerical data.
Source Data Extended Data Fig. 8 Numerical data.
Source Data Extended Data Fig. 10 Numerical data.

## Code availability

Scripts used in this study are deposited on GitHub at https://github.com/NavarroVettiger/Navarro-et-al_2022.
