# Supplementary material for: Cell wall synthesis and remodelling dynamics determine division site architecture and cell shape in Escherichia coli
Source: Nat Microbiol. 2022 Sep 12;7(10):1621–34. doi: 10.1038/s41564-022-01210-z (PMC9519445; doi:10.1038/s41564-022-01210-z)
Supplement: Supplementary file 1 — Supplementary Figs. 1–10, Tables 1–6 and Video legends 1–8. [file 41564_2022_1210_MOESM1_ESM.pdf]

nature portfolio

https://doi.org/10.1038/s41564-022-01210-z

Supplementary information

Cell wall synthesis and remodelling dynamics determine division site architecture and cell shape in *Escherichia coli*

In the format provided by the authors and unedited

Supplemental Material For:

Cell wall synthesis and remodeling dynamics determine division site architecture and cell shape in *Escherichia coli*

Authors

Paula P. Navarro[1,2,†], Andrea Vettiger[3,†], Virly Y. Ananda[1], Paula Montero Llopis[4], Christoph Allolio[5], Thomas G. Bernhardt*[,3,6] , and Luke H. Chao*[,1,2]

Affiliations

[1] Department of Molecular Biology, Massachusetts General Hospital, Boston, USA

[2] Department of Genetics, Blavatnik Institute, Harvard Medical School, Boston, USA

[3] Department of Microbiology, Blavatnik Institute, Harvard Medical School, Boston, USA

[4] MicRoN Core, Harvard Medical School, Boston, USA

[5] Faculty of Mathematics and Physics, Mathematical Institute, Charles University, Prague, Czech Republic

[6] Howard Hughes Medical Institute, Harvard Medical School, Boston

[†] These authors contributed equally, and sequence was determined alphabetically.

* To whom correspondence should be addressed (order determined alphabetically)

Thomas G. Bernhardt

Department of Microbiology, Harvard Medical School, Boston, USA

e-mail: thomas_bernhardt@hms.harvard.edu

Luke H. Chao

Department of Molecular Biology, Massachusetts General Hospital, Boston, USA

Department of Genetics, Harvard Medical School, Boston, USA

e-mail: chao@molbio.mgh.harvard.edu

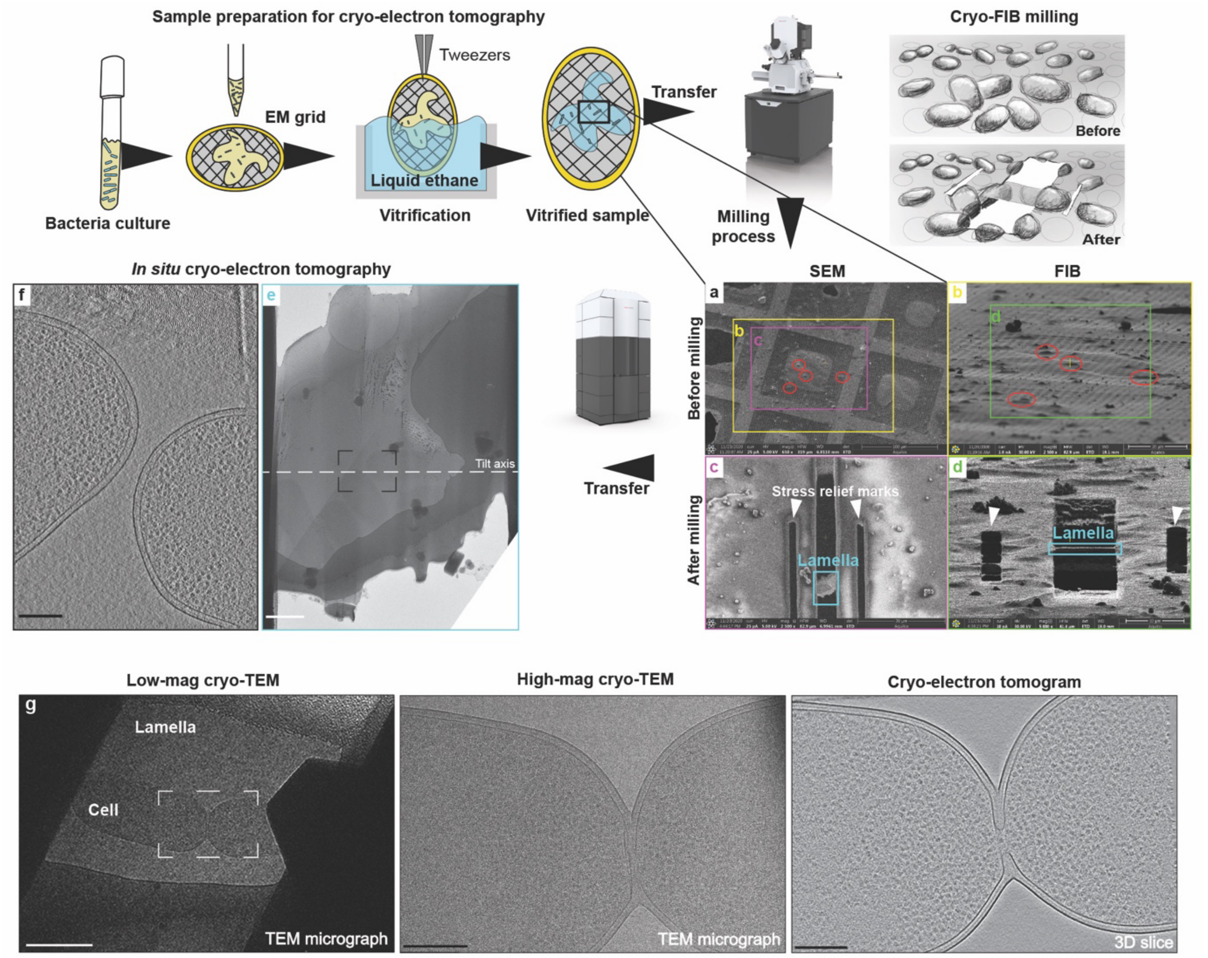

Supplementary Figure S1: Cryo-FIB / cryo-ET pipeline utilized in this study. Schematic cartoons showing the steps in sample preparation for cryo-ET. In brief, bacteria are grown to OD_{600} = 0.3 and applied onto an EM grid for vitrification in liquid ethane[86]. Cryo-EM grids are kept in liquid nitrogen until transfer into the cryo-FIB microscope for milling. An illustration shows the result of milling vitrified bacteria distributed onto the holey carbon film on the mesh EM grid. (a-d) Images taken from the Aquilos Thermo Fisher Scientific graphical user interface during cryo-FIB milling performance. (a) Target bacteria (red circles) are first identified by SEM, e^- beam. Yellow box indicates region visualized in (b) and magenta box indicates the area visualized in (c). (b) Corresponding FIB, ion Ga^+ beam, view (52° with respect to the e^- beam[58]) of the targeted grid square in (a) (yellow box). Green box indicates region visualized in (d). (c) SEM view of the same region shown in (a) and (b) after platinum deposition and milling. Cyan box indicates obtained lamella shown in (e). (d) FIB view of region shown in (b) (green box) after platinum deposition and milling. Scale bars in (a-d) are indicated on each image. After milling, cryo-EM grids containing bacterial lamellae are transferred into a TEM microscope. (e) Low magnification TEM 2D image of the lamella shown in (c-d), cyan box. Dashed black box indicates target region for cryo-ET acquisition. Dashed white line indicates the tilt axis for cryo-ET data acquisition. (f) 3D slice of the cryo-electron tomogram obtained from 3D reconstruction of aligned cryo-ET tilt series acquired in (e) (dashed black box). Scale bars: e = 1000 nm; f = 200 nm. (g) A representative lamella from a wild-type *E. coli* cell imaged at indicated imaging conditions. White box highlights region for corresponding high-magnification acquisition. Scale bars = 1 μm (low magnification); 200 nm (high magnification and cryo-electron tomogram).

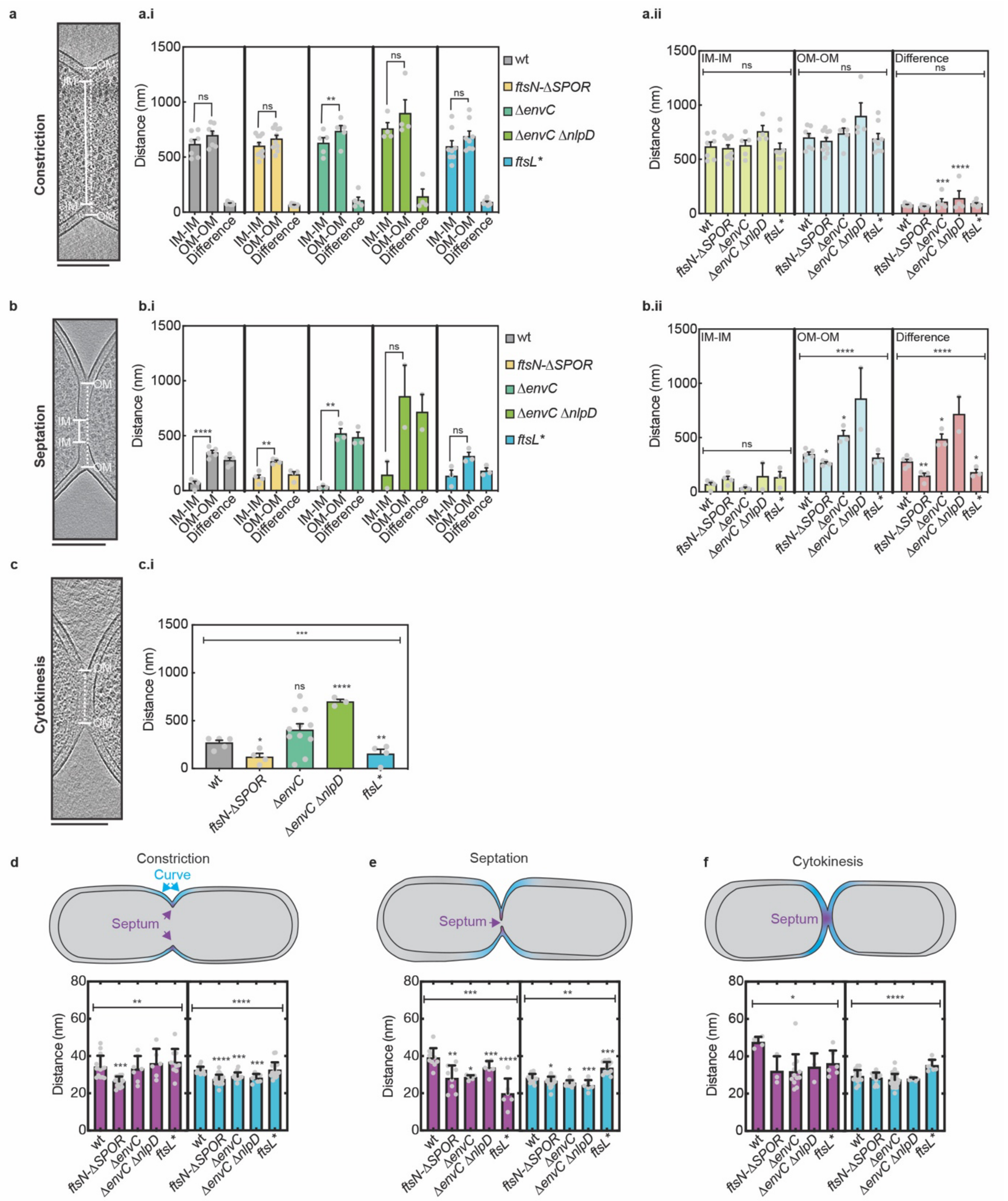

Supplementary Figure S2: Distance measurements in cryo-ET data of dividing *E. coli* cells. Three dimensional slices visualizing the division site during (a) constriction, (b) septation and (c) cytokinesis. Dashed white line indicates OM-OM distance and white bold line indicates IM-IM distance. (a.i-c.i) Measured distances in nm of IM-IM, OM-OM and the difference between these distances at (a.i) constriction (N = 7 (wt); 10 (*ftsN*-Δ*SPOR*); 5 (Δ*envC*); 4 (Δ*nlpD* Δ*envC*); 8 (*ftsL**) images), (b.i) septation (N = 5 (wt); 4 (*ftsN*-Δ*SPOR*); 3 (Δ*envC*); 2 (Δ*nlpD* Δ*envC*); 3 (*ftsL**) images), and (c.i) cytokinesis (N = 5 (wt); 4 (*ftsN*-Δ*SPOR*); 11 (Δ*envC*); 3 (Δ*nlpD* Δ*envC*); 4 (*ftsL**) images). (a.ii-b.ii) Scale bars = 200 nm. All data are expressed as mean + SEM. (d-f) Schematic representing the division stages color-coded at where periplasmic width was measured. Thirty euclidean distances were measured per region (see Methods), N values for each region per stage are: (d) at septum (N = 14 (wt); 16 (*ftsN*-Δ*SPOR*); 7 (Δ*envC*); 6 (Δ*nlpD* Δ*envC*); 10 (*ftsL**)); at curve (N = 15 (wt); 27 (*ftsN*-Δ*SPOR*); 13 (Δ*envC*);

8 (Δ*nlpD* Δ*envC*); 19 (*ftsL**)); (e) at septum (N = 10 (wt); 8 (*ftsN*-Δ*SPOR*); 6 (Δ*envC*); 6 (Δ*nlpD* Δ*envC*); 6 (*ftsL**)); at curve (N = 19 (wt); 23 (*ftsN*-Δ*SPOR*); 12 (Δ*envC*); 6 (Δ*nlpD* Δ*envC*); 12 (*ftsL**)); (f) at septum (N = 4 (wt); 3 (*ftsN*-Δ*SPOR*); 12 (Δ*envC*); 3 (Δ*nlpD* Δ*envC*); 6 (*ftsL**)); at curve (N = 16 (wt); 12 (*ftsN*-Δ*SPOR*); 39 (Δ*envC*); 6 (Δ*nlpD* Δ*envC*); 7 (*ftsL**)). All analyzed data points are displayed, bar represents mean + SD.

Significance was tested using unpaired t-test with Welch correction for paired groups when data followed gaussian distribution and Mann-Whitney when data did not follow gaussian distribution and tested relative to wild-type. Significance difference among all groups compared was tested using Welch ANOVA and Brown-Forsythe when data followed gaussian distribution and Kruskal-Wallis test when data did not follow gaussian distribution. In (a.ii) difference plot, statistical significance is shown based on F-test statistics. Ns = non-significant, * = $p < 0.05$, ** = $p < 0.01$, *** = $p < 0.001$, **** = $p < 0.0001$.

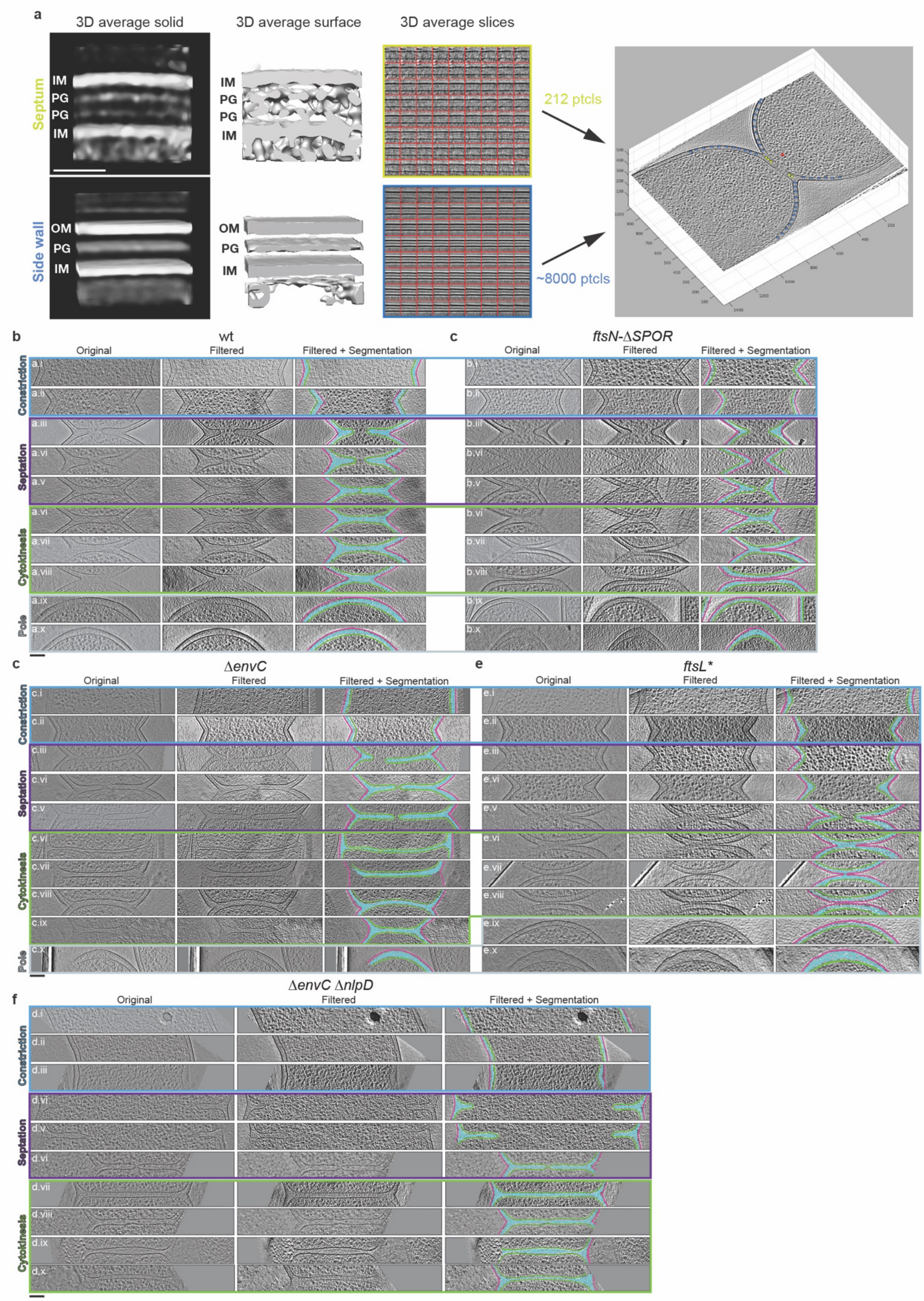

Supplementary Figure S3: Subtomogram averaging (STA), NAD filtering and segmentation of the cell envelope of *E. coli*. (a) STA 3D structure of the cell envelope at the septum and sidewall are displayed in Chimera using solid and surface rendering. 3D slices of averages are shown. 212 particles contributed to the septum average while 8072 particles from N = 5 tomograms contributed to the sidewall average. Blue dots plotted on a tomogram

slice represent particles that contributes to ‘sidewall’ and yellow dots represent particles contributing to septum average. Scale bar = 40 nm. In the tomogram rendering, 100 pixels blocks in the cartesian axes correspond to 102.6 nm. (b-f) Gallery of corresponding zoom-in summed projected central slices of cryo-electron tomograms visualizing the indicated division mutants. First column shows original image, second column shows filtered image, and third column shows filtered image with segmentation layers indicating IM = green, PG = cyan and OM = magenta. A full cryo-ET gallery can be found in Supplementary Figure 5. A complete overview of the number of tomograms is reported in Tables S2-S3. Scale bars = 100 nm.

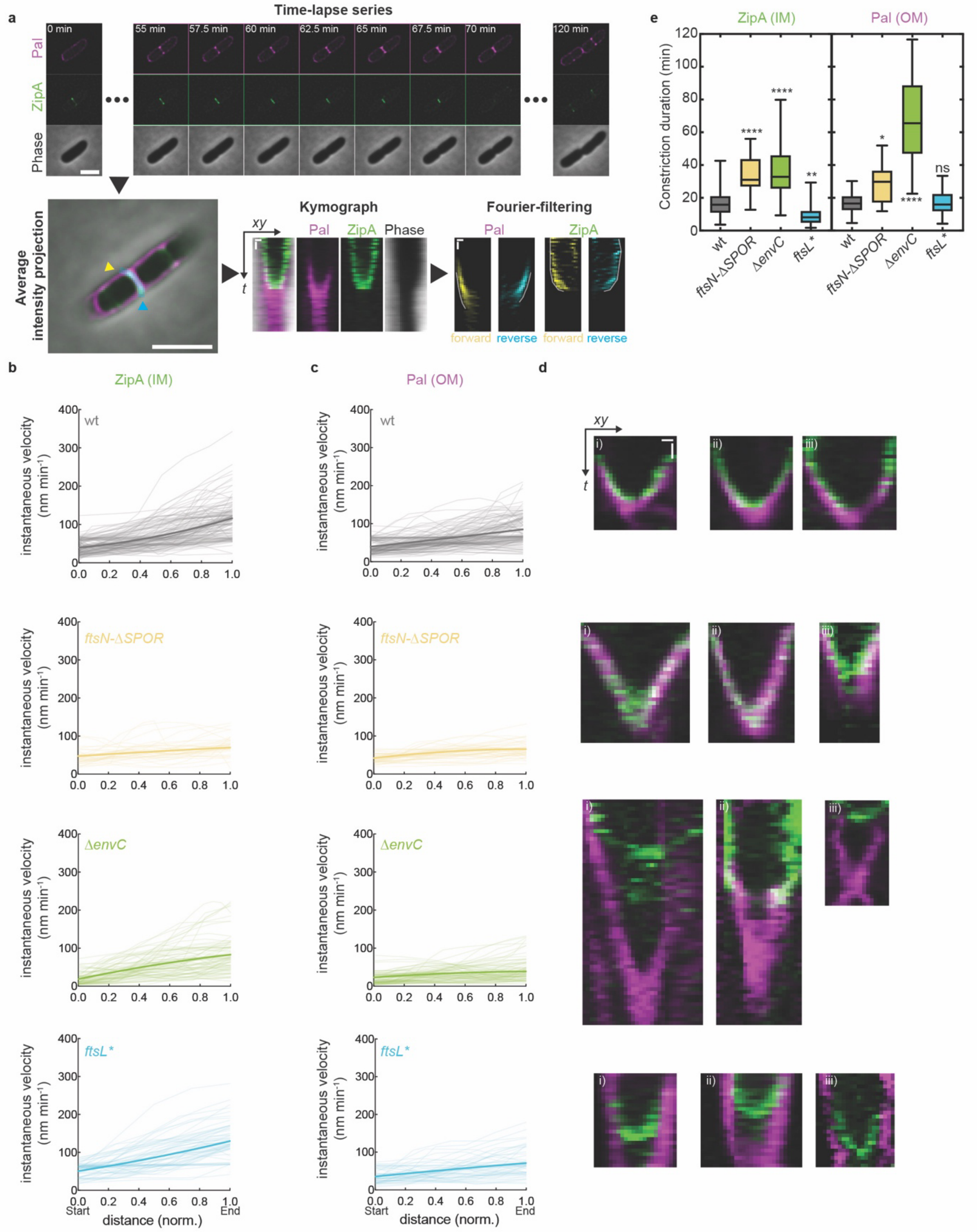

Supplementary Figure S4: Measuring cell envelope constriction from kymograph data. (a) Schematic representation of workflow for the generation of kymographs using *Kymoclear* and *KymogrphaDirect* software. Instantaneous constriction velocity for (b) IM (ZipA-sfGFP) and (c) OM (Pal-mCherry) are plotted against normalized cell width. Bold lines show second order polynomial fits as in Figure 2e-f. N = 150 cells (wt); N = 48 (*ftsN*-Δ*SPOR*); N = 81 (Δ*envC*)**;** N = 68 (*ftsL**) kymographs. (d) Additional examples of cell envelope constriction kymographs for the corresponding strains in panels b-c. (e) Duration of IM (left) and OM (right) constriction was derived from kymograph measurements. Data are represented as boxplots. The line represents median; error bars depict Min-Max range. The significance of differences were tested relative to wild-type by one-way ANOVA with Dunnett's correction for multiple

comparisons; ns = non-significant ($p = 0.99$), * = $p < 0.05$, *** = $p < 0.001$, **** = $p < 0.0001$; N = 150 cells (wt); N = 48 (*ftsN*-Δ*SPOR*); N = 81 (Δ*envC*); N = 68 (*ftsL**). Scale bar = 2 µm, in kymographs = 200 nm horizontal and 10 min vertical.

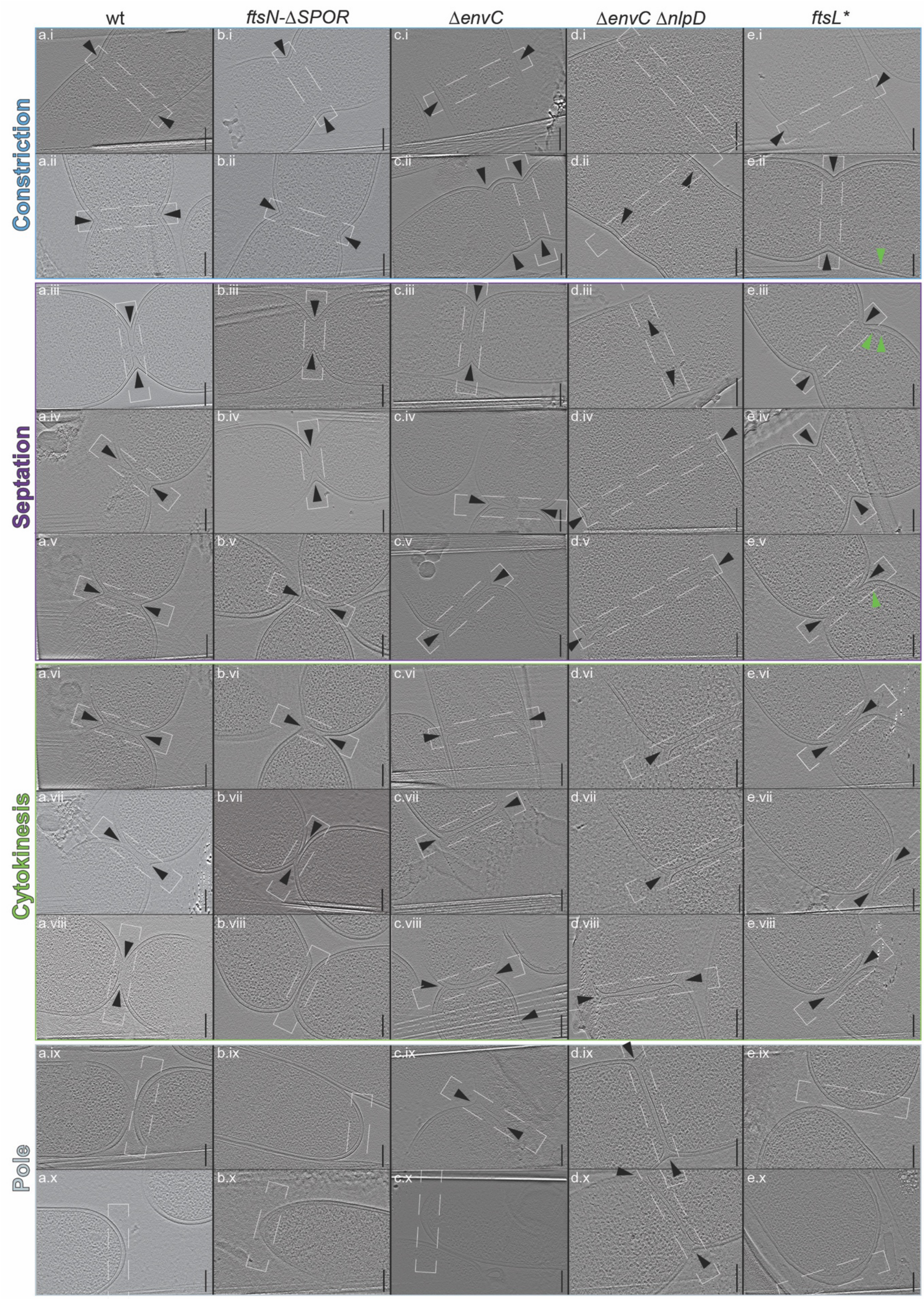

Supplementary Figure S5: Cell division and polar morphology of *E. coli* viewed by cryo-ET. Gallery of summed projected central slices of cryo-electron tomograms visualizing the indicated division mutants. Black arrowhead = division site; green arrowhead = envelope bulging. Dashed white box indicates corresponding zoom-in region show in Supplementary

Figure 3. A complete overview of number of tomograms is reported in Tables S2-S3. Scale bars = 200 nm.

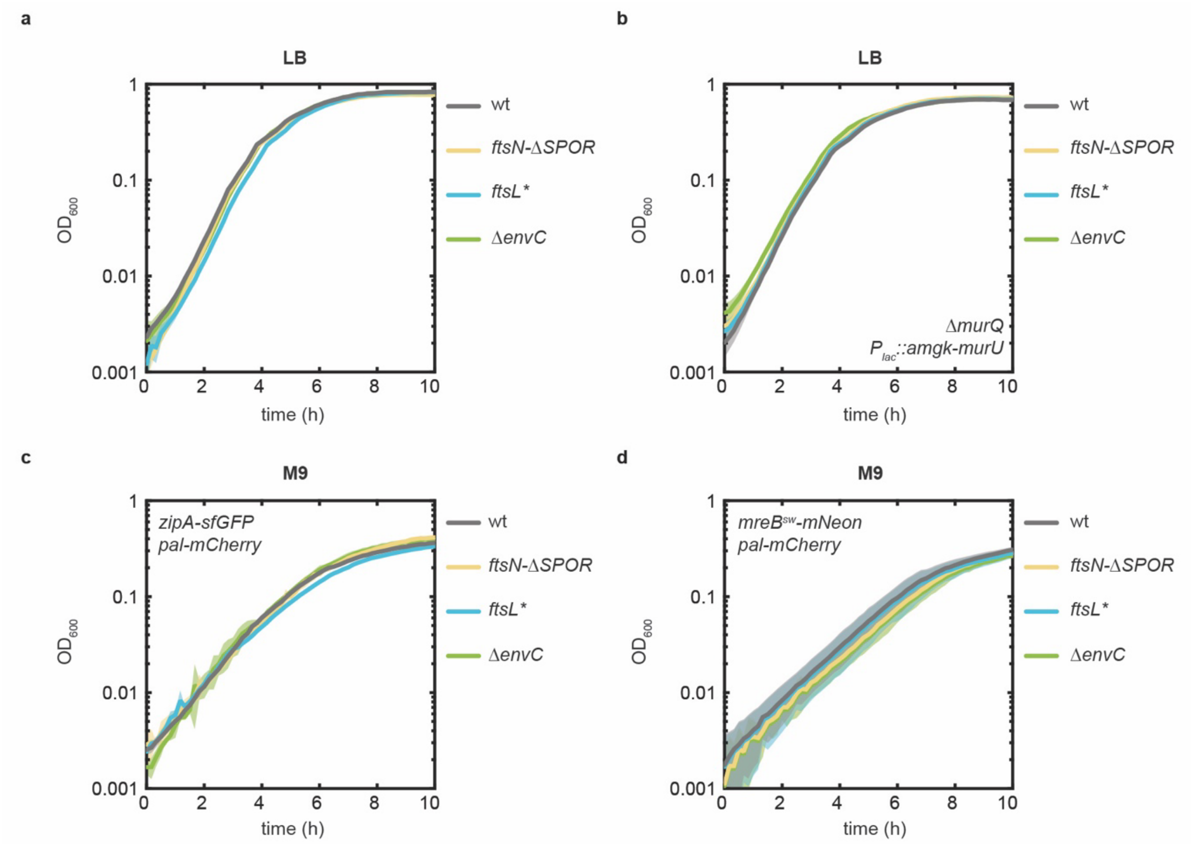

Supplementary Figure S6: Measuring bulk growth rates of *E. coli* cell division mutants analyzed in this study. Growth curves were measured in biological triplicates by OD_{600} readings in a 96-well plate reader at 30˚C. Data is represented as mean ± SD. (a) Untagged strains used for cryo-ET and FDAA labeling as well as (b) Δ*murQ* mutants expressing *amgK* and *murU* for MurNAc-alykyne labeling experiments were grown in LB. Cells harboring fluorescent fusion proteins for live-cell imaging of (c) cell envelope constriction or (d) MreB tracking were grown in M9 medium supplemented with 0.2% glucose and casamino acids.

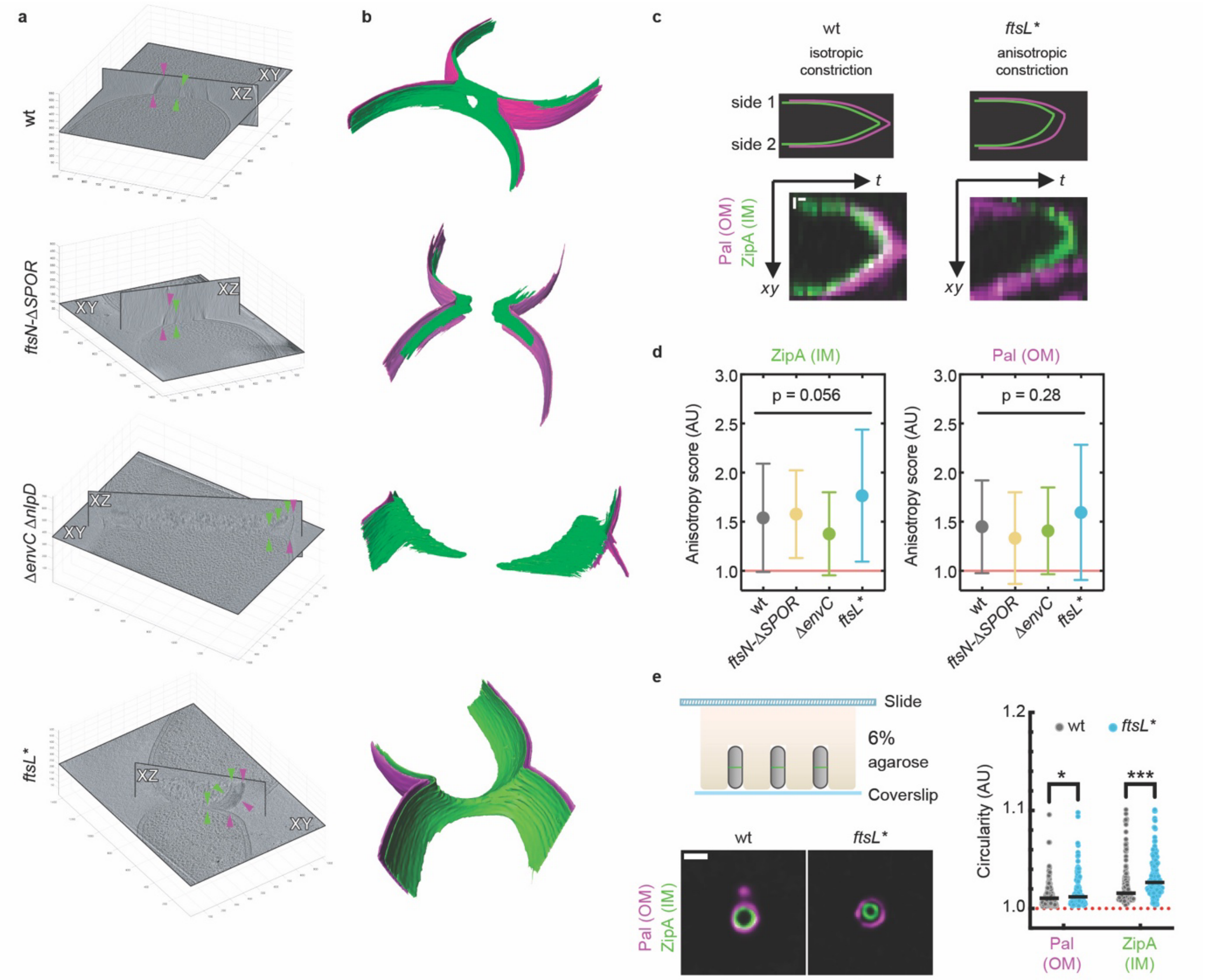

Supplementary Figure S7: A hyperactivated divisome leads to anisotropic cell envelope constriction. (a) Orthogonal views of XZ and XY slices of 3D cryo-electron tomograms of the indicated division mutants. Magenta and green arrowheads indicate OM and IM, respectively. 3D volumes are displayed in cartesian 3D grids with axes indicating the dimensions in pixels. For WT 100 pixels = 102.6 nm, and for *ftsL** 100 pixels = 110.3 nm. (b) Corresponding 3D surface segmentation renderings of OM (magenta) and IM (green) are shown on the right. (c) Schematic overview of a theoretical kymograph for an isotropic (left) and anisotropic (right) constriction of the cell envelope. Representative examples form 3 biological replicates for wild-type (left) and *ftsL** (right) are provided. Scale bars: 200 nm (vertical); 5 min (horizontal). (d) An anisotropy score was calculated by taking the ratio of the constriction velocity from both sides of the cell. Red line (= 1) indicates a perfectly isotropic cell envelope constriction process. Data are represented as mean ± SD, Kruskal-Wallis with Dunn's correction for multiple comparisons among all values was calculated, exact p values are shown. N = 65 (wt); 24 (*ftsL**); 23 (*ftsN-ΔSPOR*); 44 *(ΔenvC*) constriction were analyzed (e) Cells were vertically immobilized using small micro pillars imprinted into agarose pads, allowing to image the cell division site along its long axis. Representative example of the cell envelope position in vertically imaged wt and *ftsL** cells. Scale bar = 2 µm. Circularity was quantified using *Morphometrics*. Red line (circularity = 1) indicates a perfect circle. Black line indicates median. Two-way ANOVA with Sidak's multiple comparison test; * = $p < 0.05$; *** = $p < 0.001$. N = 132 (wt); 172 (*ftsL**) cells imaged in three biological replicates.

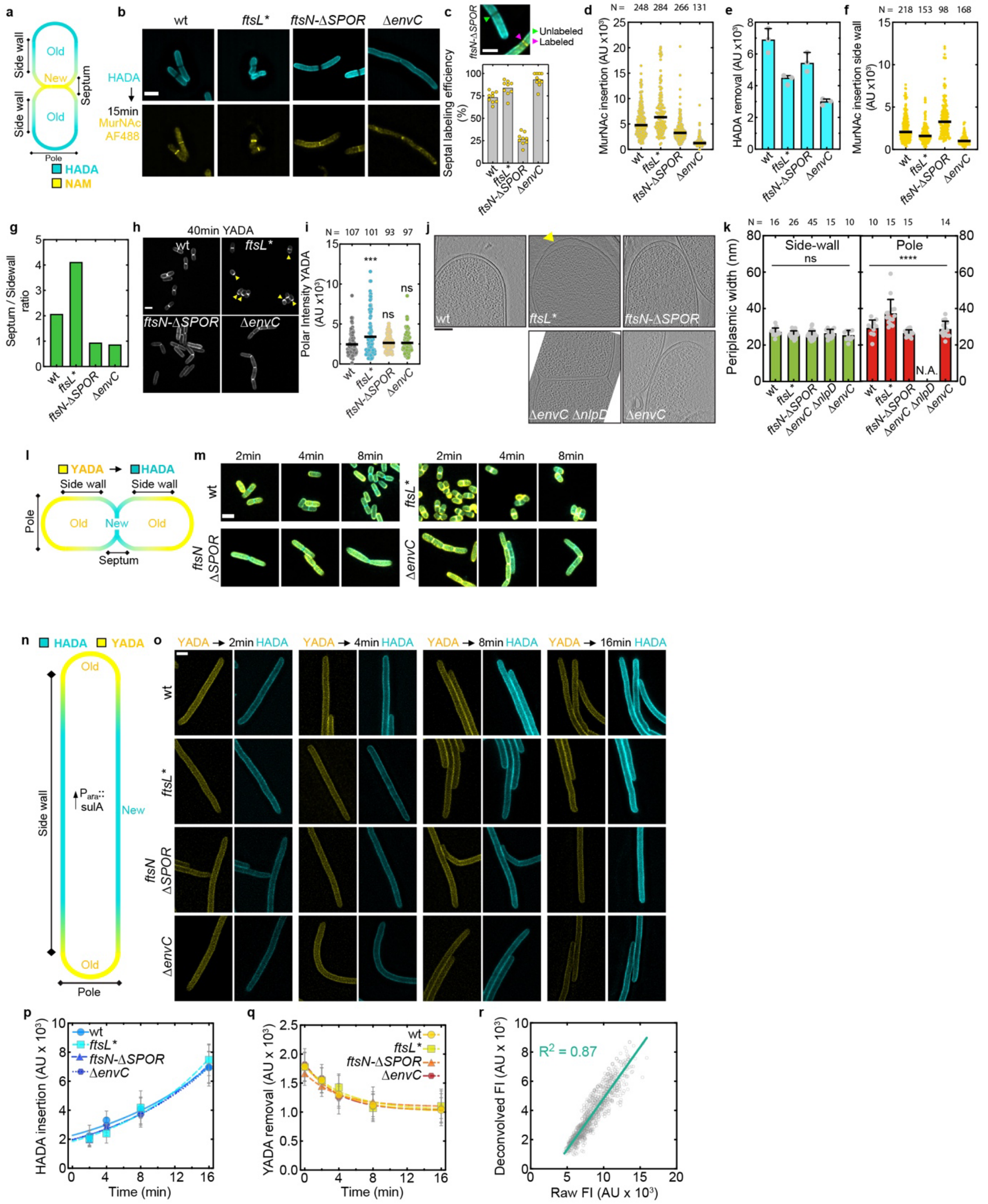

Supplementary Figure S8: Cell wall synthesis and hydrolysis measurements. (a) New and old cell wall material were detected with Alexa488 labeled MurNAc-alykyne (yellow) or HADA (blue), respectively. (b) Representative images from labeling. (c) Label incorporation at the division site. Bars show the mean and dots show the average from 9 different images. *ftsN-ΔSPOR* labeling example. (d) Rate of sPG synthesis (line = median). (e) Septal PG hydrolysis. Bar represents median + 95 % confidence interval; points indicate the average of three biological replicates. (f) Side wall labeling (line = median). (g) sPG and sidewall synthesis ratio. (h) Representative YADA labeling pattern. Yellow arrow heads indicate polar label accumulation. (i) Average polar YADA fluorescence. Black line indicates mean, one-way ANOVA with Dunnett's correction for multiple comparison. Significant differences are relative

to wild-type; ns = non-significant ($p = 0.76$), $p < 0.00$. (j) Summed projected central 3D slices through tomograms of poles. Yellow arrowhead indicates enlarged periplasm. (k) Periplasm thickness in cryo-ET data at the side wall (green) and pole (red) (mean + SD). Thirty Euclidean distances were measured per region. One-way ANOVA (sidewall) and Kruskal-Wallis test (pole); significance was tested among all groups within each region; ns = non-significant ($p = 0.91$), **** = $p < 0.0001$, N.A. = not applicable. (l) Labeling patterns observed for the pulse-chase. (m) Sum-projection of deconvolved images after HADA pulses as shown in Figure 3b. (n) Expected labeling patterns for cells expressing *sulA*. New and old wall is labeled with HADA (blue) and YADA (yellow), respectively. (o) Representative images of indicated strains after HADA pulses. Mean sidewall fluorescence intensity for (p) HADA and (q) YADA fit to a (p) Malthusian exponential function or (q) one phase exponential decay. Mean fluorescence intensity ± one SD is shown, N = 360 cells each. (r) Fluorescence intensity (p-q) was measured in original non-deconvolved (raw) SUM projections. Linear regression shows strong positive ($R^2 = 0.87$) correlation to deconvolved fluorescence intensity values. N = 1035 values. Scale bars = 2μm (fluorescence) or 200 nm (cryo-ET).

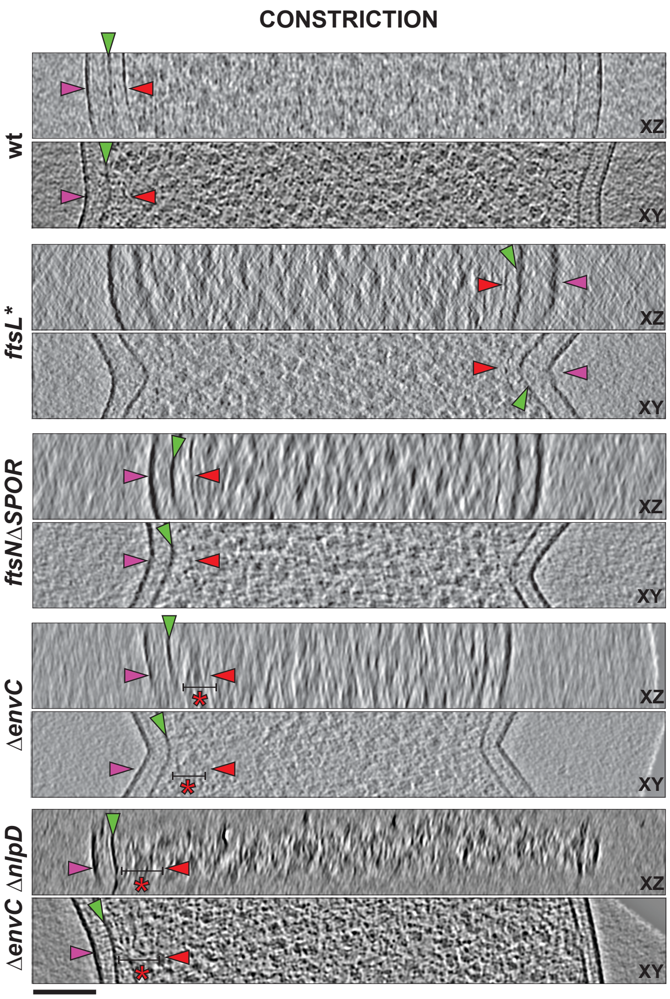

Supplementary Figure S9: Z-ring views during constriction in cryo-electron tomograms of *E. coli*. Summed projections of 10 slices of XZ and XY views during constriction of indicated strains. Representative examples of all strains are shown. Green arrowheads indicate IM, magenta arrowheads indicate OM, red arrowheads indicate cytoskeletal ring and red asterisks indicate zones of diffuse signal. Note, Z-ring signal is weaker in Δ*envC* mutants due to issues with Z-ring condensation as shown by fluorescence microscopy (Fig. 4h-j) Scale bar = 100 nm.

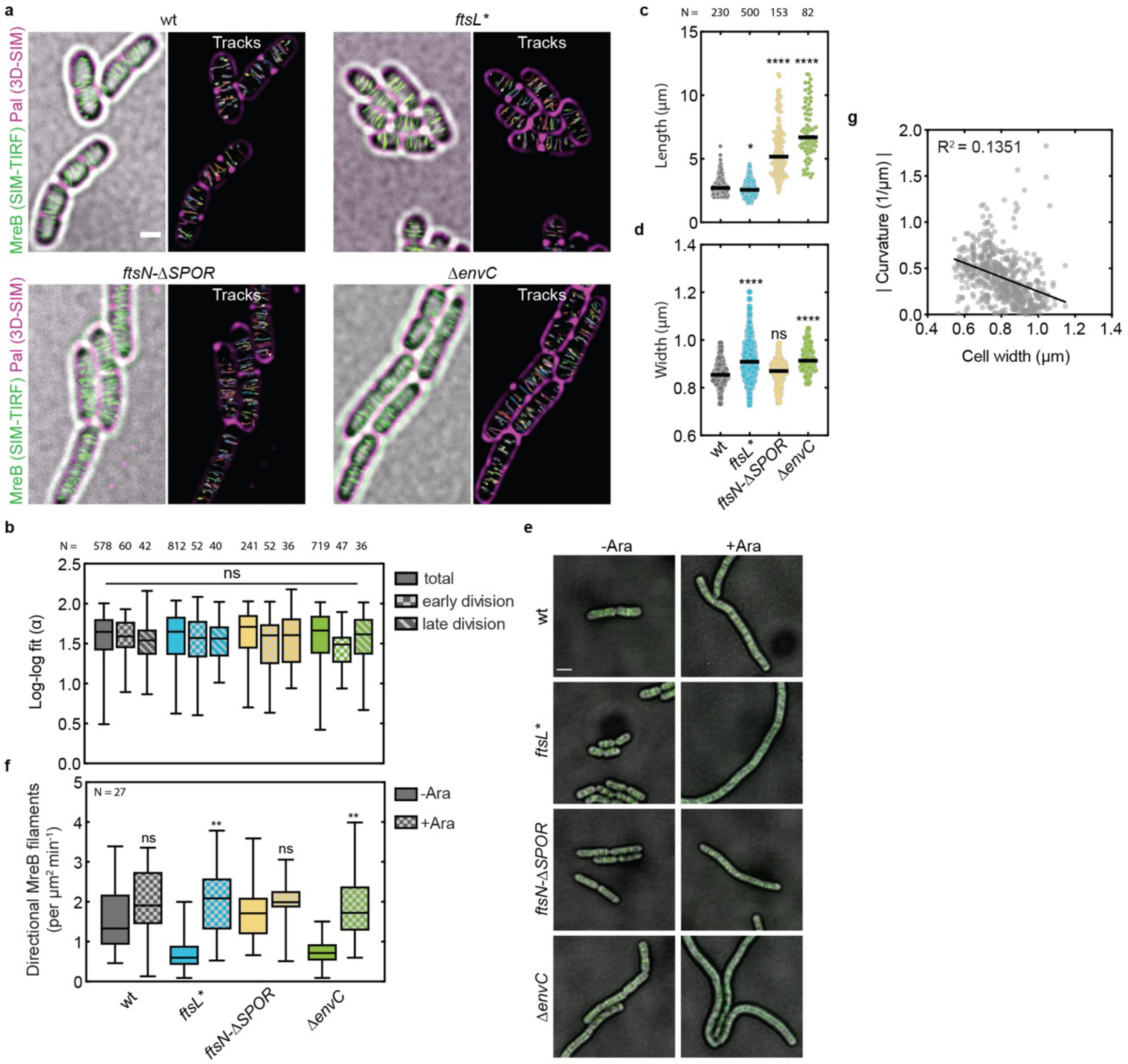

Supplementary Figure S10: The balance between elongation and division affects cell morphology. (a) MreB-sw-mNeonGreen dynamics were followed by SIM-TRIF microscopy for 3 min at 3 s acquisitions per frame in indicated mutants. Time-lapse series was sum-projected and overlayed over a 3D-SIM Pal-mCherry and brightfield reference image. Larger fields of view are shown as compared to Figure 5G and are representative from three biological replicates. Bar = 1 μm (b) Slopes of MSD curves (α) were analyzed following log-log fit to $log\,[MSD]$ versus $log\,[t]$ using the MATLAB class msdanalyzer. Particles displaced by diffusive motion are characterized by a slope of their $log\,[MSD]$ = 1, while transported particles have slopes of 2 and constrained particles display slopes < 1. No significant difference (ns = non-significant, p = 0.0693; Kruskal Wallis test with Dunn's correction for multiple comparisons) in the slopes of MSD curves were found indicating that MreB is displaced at a similar rate and manner in all strains. Box plot error bars displaying Min-Max range of values, blackline represents median. Tracks fit to $log\,[MSD]\ log\,[t]$ with $R^2 \leq 0.95$ (c) Cell length and (d) width was measured from three independent biological replicates for the indicated mutants using *Morphometrics*. Line represents median. Differences in significances were tested relative to wild-type using Kruskal-Wallis one-way ANOVA with Dunnett's correction for multiple comparisons; * = p < 0.05, **** = p < 0.0001, ns = non-significant, p = 0.69. (e) MreB dynamics were imaged as in (a). Representative temporal SUM projections of MreB-mNeon trajectories were overlayed to brightfield images. Bar = 2 μm. (f) Directionally moving MreB tracks were filtered by MSD analysis (see Methods) and represented as boxplots (line indicating median; error bars depict Min-Max range) and normalized by cell area.

Significance in each group was tested against non-filamented control (-Ara) by two-sided unpaired t-test; ** $p < 0.01$, ns = non-significant, wt $p = 0.053$, *fstN-ΔSPOR* = 0.253. (g) Constriction curvature values of wild-type cells are plotted against division site width in 566 cells. Linear regression ($R^2 = 0.135$) indicates the negative correlation between cell width at the division site and constriction angle.

Supplementary Table 1. Summary of data acquisition and image processing for cryo-ET data in this study.

Sample		wt	*ftsL**	*ftsN-ΔSPOR*	*ΔenvC ΔnlpD*	*ΔenvC*
**Cryo-FIB milling**	**Microscope**	Aquilos Cryo-FIB, FEI – Thermo Fisher Scientific	Aquilos Cryo-FIB, FEI – Thermo Fisher Scientific	Aquilos Cryo-FIB, FEI – Thermo Fisher Scientific	Aquilos Cryo-FIB, FEI – Thermo Fisher Scientific	Aquilos Cryo-FIB, FEI – Thermo Fisher Scientific
**Acquisition settings**	**Microscope**	Titan Krios Gi3 FEI, Thermo Fisher Scientific	Titan Krios Gi3 FEI, Thermo Fisher Scientific	Titan Krios Gi3 FEI, Thermo Fisher Scientific	Titan Krios Gi3 FEI, Thermo Fisher Scientific	Titan Krios Gi3 FEI, Thermo Fisher Scientific
	**Voltage (KeV)**	300	300	300	300	300
	**Detector**	Gatan K3 IS	Gatan K3 IS	Gatan K3 IS	Gatan K3 IS	Gatan K3 IS
	**Energy filter**	Gatan BioQuantum K3	Gatan BioQuantum K3	Gatan BioQuantum K3	Gatan BioQuantum K3	Gatan BioQuantum K3
	**Slit width (eV)**	20	20	20	20	20
	**Super-resolution mode**	Yes	Yes	Yes	Yes	Yes
	**Å/pixel**	1.282/1.379*	1.379	1.379	1.282/1.379* and 1.096**	1.379
	**Defocus (µm)**	-3.5 to -5.0	-3.5 to -5.0	-3.5 to -5.0	-3.5 to -5.0	-4 to -5.0
	**Acquisition scheme**	-70/70, 2°, Dose-symmetric	-70/70, 2°, Dose-symmetric	-70/70, 2°, Dose-symmetric	-70/70, 2°, Dose-symmetric	-70/70, 2°, Dose-symmetric
	**Total dose**	~90 - 120	~90 - 180	~90 - 180	~90 - 120	~90 - 190
	**Dose rate (e-/Å/sec)**	~ 1.5 - 3	~ 1.5 - 3	~ 1.5 - 3	~ 1.5 - 3	~ 1.5 - 3
	**Frame number**	4 - 6	4 - 6	4 - 6	4 - 6	4 - 6
	**Number of tomograms**	22	21	27	8	31
**Image processing**	**Frame alignment and dose weighting**	*framealign*, IMOD	*framealign*, IMOD	*framealign*, IMOD	*framealign,* IMOD	*framealign,* IMOD
	**Tilt series alignment**	IMOD/*Dynamo*	IMOD	IMOD	IMOD	IMOD
	**WBP**	IMOD	IMOD	IMOD	IMOD	IMOD
	**Filtering**	IMOD/*Dynamo*/ Amira	IMOD/*Dynamo*/ Amira	IMOD/*Dynamo*/ Amira	IMOD/*Dynamo*/ Amira	IMOD/*Dynamo*/ Amira
	**3D-segmentation**	Amira	Amira	Amira	Amira	Amira
	**3D-rendering**	*Dynamo*/Amira	*Dynamo*/Amira	*Dynamo*/Amira	*Dynamo*/Amira	*Dynamo*/Amira

*Data was acquired in two different FEI Titan Krios (Thermo Fisher Scientific).
**Data was acquired at two different magnifications (35kx and 42kx).

Supplementary Table 2. Statistics of cryo-FIB / cryo-ET acquisition.

Sample	wt	*ftsN-ΔSPOR*	Δ*envC*	Δ*envC* Δ*nlpD*	*ftsL**	Total
**N***	22	27	31	8	21	**109**
**Grids for cryo-FIB**	13	4	3	4	6	**30**
**Lamellae/grid (total # lamellae)**	6 (78)	5.25 (21)	5 (15)	4.5 (18)	5.7 (34)	**5.53 (166)**
**Grids imaged by TEM**	13	4	3	3	5	**28**
**Imaged lamellae**	18	11	14	3	11	**57**
**Cells/lamella (total # cells)**	2.61 (47)	5.27 (58)	7 (98)	5 (15)	1.9 (21)	**4.2 (239)**
**Tomogram/lamella**	1.17	2.36	2.21	2.67	1.72	**1.8**

*Number of imaged cells equals number of tomograms.

Supplementary Table 3. Summary of cryo-FIB / cryo-ET.

Stage	wt	*ftsN-ΔSPOR*	Δ*envC*	Δ*envC* Δ*nlpD*	*ftsL**	Total
**Constriction**	7	10	3	2	6	**28**
**Septation***	5	4	3	2	1	**15**
**Cytokinesis****	1	2	12	3	1	**19**
**Pole / Body**	9	11	13	1	13	**47**
**Total**	**22**	**27**	**31**	**8**	**21**	**109**

*Cells in tomograms categorized as septation stage present IM. As moving in Z across these tomograms, cytokinesis stage can be also visualized, complete IM scission. But to avoid confusion, we categorized one stage per tomogram.
**Cells in tomograms categorized as cytokinesis stage do not present IM connection across the whole tomogram.

Supplementary Table 4. Strains used in this study.

Strain	Genotype[a]	Relevant features	Used for	Source/Reference[b]
TB28	*rph1 ilvG rfb-50 ΔlacIZYA<>frt*	lacZ- MG1655, wild type	cryoET / FDAA	87
TB44	*TB28 ΔenvC<>frt*	*envC* deletion		88
TB156	*TB28 ΔenvC<>frt ΔnlpD::aph*	*nlpD* and *envC* double deletion, Kan^R		34
TT154	*TB28 ftsN(1-243)-TAA<>frt*	*ftsN-ΔSPOR* deletion		47
MT10	*TB28 ftsL(E88K)*	*ftsL** allele		26
AV203	*TB28, pNP146*	SulA expression		
AV204	*TT154, pNP146*	*ftsN-ΔSPOR* deletion, SulA expression		
AV205	*TB44, pNP146*	*envC* deletion, SulA expression		
AV206	*MT10, pNP146*	*ftsL** allele, SulA expression		
AV92	*TB28 murQ::aph, pCF436*	*murQ* deletion	NAM	P1(CF491)[83] x TB28
AV93	*TB28 ftsN(1-243)-TAA<>frt murQ::aph, pCF436*	*ftsN-ΔSPOR* deletion in Δ*murQ*		P1(CF491)[83] x TT154
AV94	*TB28 ΔenvC<>frt murQ::aph, pCF436*	Δ*envC* deletion in Δ*murQ*		P1(CF491)[83] x TB44
AV125	*TB28 ftsL(E88K) murQ::aph, pCF436*	*ftsL** allele in Δ*murQ*		P1(CF491)[83] x MT10
AV211	*AV92, pCF436, pNP146*	SulA expression		
AV212	*AV93, pCF436, pNP146*	*ftsN-ΔSPOR* deletion in Δ*murQ,* SulA expression		
AV213	*AV94, pCF436, pNP146*	Δ*envC* deletion in Δ*murQ,* SulA expression		
AV214	*AV125, pCF436, pNP146*	*ftsL** allele in Δ*murQ,* SulA expression		
AV53	*TB28 attHK022 P_{lac}::zipA-sfGFP bla pal-mCherry cat*	GFP-IM/mCherry-OM, Amp^R, Cam^R	Live-cell imaging	P1(MG11) x TB28(attHKTB225)[34]
AV67	*AV53 ftsN(1-243)-ΔSPOR::aph*	*ftsN-ΔSPOR* deletion in GFP-IM/mCherry-OM, Amp^R, Cam^R, Kan^R		P1(NP279)[47] x AV53
AV76	*AV53 ΔenvC::aph*	*envC* deletion in GFP-IM/mCherry-OM, Amp^R, Cam^R, Kan^R		P1(JW5646)[89] x AV53
AV115	*AV53 leu::TN10*	Strain intermediate for AV116, Amp^R, Cam^R, Tet^R		P1(CH43)[24] x AV53
AV116	*AV53 ftsL(E88K)*	*ftsL** allele in GFP-IM/mCherry-OM, Amp^R, Cam^R		P1(MT10)[26] x AV115
AV134	*AV53 ftsL(E88K) ΔenvC::aph*	Δ*envC* deletion in ftsL* in GFP-IM/mCherry-OM, Amp^R, Cam^R, Kan^R		P1(JW5646)[89] x AV116

AV136	*AV53 ΔfliO::aph*	Flagellum deletion in GFP-IM/mCherry-OM, Amp^{R}, Cam^{R}, Kan^{R}		P1(JW5316)[89] x AV53
AV137	*AV53 ftsL(E88K) ΔfliO::aph*	Flagellum deletion in *ftsL** GFP-IM/mCherry-OM, Amp^{R}, Cam^{R}, Kan^{R}		P1(JW5316)[89] x AV116
AV150	*TB28 mreB'-mNeonGreen-'mreB ΔyhdE pal-mCh cat*	MreB-mNG, Pal-mCh, Cam^{R}	SIM-TIRF	P1(MG11) x AV7
AV151	*AV150 ftsL(E88K)*	*ftsL** in MreB-mNG Pal-mCh background, Cam^{R}		P1(MG11) x AV149
AV152	*AV150 ΔenvC::aph*	Δ*envC* in MreB-mNG Pal-mCh background, Cam^{R}, Kan^{R}		P1(MG11) x AV146
AV154	*AV150 ftsN(1-243)-ΔSPOR::aph*	*ftsN-ΔSPOR* in MreB-mNG Pal-mCh background, Cam^{R}, Kan^{R}		P1(MG11) x AV147
AV207	*AV150, pNP146*	MreB-mNG, Pal-mCh, Cam^{R}, SulA expression		
AV208	*AV151, pNP146*	*ftsL** in MreB-mNG Pal-mCh background, Cam^{R}, SulA expression		
AV209	*AV152, pNP146*	Δ*envC* in MreB-mNG Pal-mCh, background, Cam^{R}, Kan^{R}, SulA expression		
AV210	*AV154, pNP146*	*ftsN-ΔSPOR* in MreB-mNG Pal-mCh background, Cam^{R}, Kan^{R}, SulA expression		

[a] The KanR cassette is flanked by FLP recognition target (frt) sites for removal by FLP recombinase. An frt scar remains following removal of the cassette using FLP recombinase expressed from pCP20. Numbers in parentheses indicate the codons included in the relevant clones.

[b] Strain constructions by P1 transduction are described using the shorthand: P1(donor) x recipient. Transductants were selected on LB Kan, Tet, Cm, or minimal medium with no casamino acids plates where appropriate. Strains resulting from the removal of a drug resistance cassette using pCP20[55] are indicated as: Parental strain/pCP20.

Supplementary Table 5. Plasmids used in this study.

Plasmid	Genotype[a]	ori	Relevant features	Source/Reference[b]
pCF436	*aacC1 bla Tn7 lacIq* P_{lac}*::amgK-murU*	colE1	IPTG inducible AmgK and MurU expression	83
pNP146	*tet Para::sulA*	colE1	Arabinose inducible SulA expression	74

Supplementary Table 6. Conservation of *E. coli* divisome components.

Gene	KEGG accession number	Homologues[a] in		
		Enterobacterales	Proteobacteria	Outside proteobacteria
ftsZ	K03531	97.4 %	93.3 %	83.9 %
ftsA	K03590	96.2 %	93.9 %	80.1 %
zipA	K03528	89.7 %	36.2 %	0.3 %
ftsI	K03587	95.1 %	94.6 %	83.1 %
ftsW	K03588	95.3 %	94.7 %	87.8 %
ftsN[b]	K03591	47.5 %[2]	8.0 %[b]	0.2 %
ftsQ,L,B[c]	K03589	93.8%	68.1%	23.9 %[c]
amiA,B,C	K01448	94.5 %	88.5 %	75.8 %
envC	K22719	94.3 %	85.2 %	42.5 %
nlpD	K06194	93.1 %	67.4 %	32.8 %
pal	K03640	93.2 %	91.6 %	60.6 %
rpoB[d]	K03043	96.0 %	88.7 %	78.9 %

[a] = Homologues were identified using AnnoTree v1.2[90]. Default setting were used for all queries: 30 % amino acid identify, E value = 0.00001, 70 % subject alignment, 70% query alignment.

[b] = *ftsN* is a widespread divisome component found in most proteobacteria, albeit highly variable at the amino acid level[91]. Thus, the here reported values for enterobacterales and proteobacteria are heavily underestimating the true abundance of *ftsN* homologues.

[c] = *ftsQLB* are widespread regulatory divisome components found in most bacteria, albeit highly variable at their amino acid level. Homologues of FtsB and FtsQ called DivIC and DivIB respectively, are broadly conserved among firmicutes.

[d] = *rpoB* servers as a control for non-divisome associated protein.

Supplementary Video 1. ***In situ*** **cell division of wild-type** ***E. coli*****.** Cryo-electron tomograms of wt *E. coli*. Time-lapse series were acquired with a rate of 7 fps in the compressed format m4v for visualization purposes. Green, cyan and magenta layers indicate segmented IM, PG and OM, respectively. Scale bars = 100 nm.

Supplementary Video 2. Fluorescence live-cell imaging of cell envelope constriction in ***E. coli*** **division mutants.** Three time-lapse series of each indicated *E. coli* mutants acquired at a 2:30 min:sec acquisition interval are shown. Bacteria were imaged at 30˚C on 1 % agarose in M9 supplemented with 0.2 % casamino acids and D-glucose. Fluorescence channels (Pal-mCherry – magenta, ZipA-sfGFP – green) were deconvolved. Video was rendered at 12 fps. Scale bar = 2 µm.

Supplementary Video 3. ***In situ*** **cell division of** ***ftsN-∆SPOR*****.** Cryo-electron tomograms of *ftsN-∆SPOR* mutant. Time-lapse series were acquired with a rate of 7 fps in the compressed format m4v for visualization purposes. Green, cyan and magenta layers indicate segmented IM, PG and OM, respectively. Scale bars = 100 nm.

Supplementary Video 4. ***In situ*** **cell division of** ***∆envC ∆nlpD*****.** Cryo-electron tomograms of *∆envC ∆nlpD* mutant. Time-lapse series were acquired with a rate of 7 fps in the compressed format m4v for visualization purposes. Green, cyan and magenta layers indicate segmented IM, PG and OM, respectively. Scale bars = 100 nm.

Supplementary Video 5. ***In situ*** **cell division of** ***ftsL*******.** Cryo-electron tomograms of *ftsL** mutant. Time-lapse series were acquired with a rate of 7 fps in the compressed format m4v for visualization purposes. Green, cyan and magenta layers indicate segmented IM, PG and OM, respectively. Scale bars = 100 nm.

Supplementary Video 6. Cell wall hydrolysis contributes to Z-ring condensation. Three-dimensional maximum intensity projections rendered in Huygens (SVI) of indicated *E. coli* strain expressing Pal-mCherry (magenta) and ZipA-sfGFP (green) are shown. Video was rendered at 12 fps. Scale bar = 2µm.

Supplementary Video 7: MreB filament increase in cells with blocked cell division. Cell division was inhibited by expressing SulA using 0.2% L-arabinose. A three-minute SIM-TRIF time-lapse series of MreB-sw-mNeonGreen (green) was overlayed over a bright field reference image. Video was rendered at 12 fps. Scale bar = 2 µm.

Supplementary Video 8: MreB filaments regularly pass either through or in direct proximity of the cell division site. A three-minute SIM-TRIF time-lapse series of MreB-sw-mNeonGreen (green) was overlayed over a 3D-SIM image of Pal-mCherry (magenta) and bright field reference image. On the right-side tracking results from TrackMate are overlayed. Video was rendered at 12 fps. Scale bar = 1 µm.
