# Supplementary material for: Cell wall synthesis and remodelling dynamics determine division site architecture and cell shape in Escherichia coli
Source: Nat Microbiol. 2022 Sep 12;7(10):1621–34. doi: 10.1038/s41564-022-01210-z (PMC9519445; doi:10.1038/s41564-022-01210-z)
Supplement: Supplementary file 2 — Reporting Summary [file 41564_2022_1210_MOESM2_ESM.pdf]

nature portfolio

Corresponding author(s): Luke H. Chao; Thomas G. Bernhardt

Last updated by author(s): Oct 8, 2021

Reporting Summary

Nature Portfolio wishes to improve the reproducibility of the work that we publish. This form provides structure for consistency and transparency in reporting. For further information on Nature Portfolio policies, see our Editorial Policies and the Editorial Policy Checklist.

Statistics

For all statistical analyses, confirm that the following items are present in the figure legend, table legend, main text, or Methods section.

n/a	Confirmed	
☐	☒	The exact sample size (*n*) for each experimental group/condition, given as a discrete number and unit of measurement
☐	☒	A statement on whether measurements were taken from distinct samples or whether the same sample was measured repeatedly
☐	☒	The statistical test(s) used AND whether they are one- or two-sided<br>*Only common tests should be described solely by name; describe more complex techniques in the Methods section.*
☒	☐	A description of all covariates tested
☐	☒	A description of any assumptions or corrections, such as tests of normality and adjustment for multiple comparisons
☐	☒	A full description of the statistical parameters including central tendency (e.g. means) or other basic estimates (e.g. regression coefficient) AND variation (e.g. standard deviation) or associated estimates of uncertainty (e.g. confidence intervals)
☐	☒	For null hypothesis testing, the test statistic (e.g. *F*, *t*, *r*) with confidence intervals, effect sizes, degrees of freedom and *P* value noted<br>*Give P values as exact values whenever suitable.*
☒	☐	For Bayesian analysis, information on the choice of priors and Markov chain Monte Carlo settings
☒	☐	For hierarchical and complex designs, identification of the appropriate level for tests and full reporting of outcomes
☒	☐	Estimates of effect sizes (e.g. Cohen's *d*, Pearson's *r*), indicating how they were calculated

Our web collection on statistics for biologists contains articles on many of the points above.

Software and code

Policy information about availability of computer code

Data collection	SerialEM 3.2 (open source, cited) and Thermo Fisher Scientific Tomography v5.3.0 (Thermo Fisher Scientific) for operation of electron microscopes (refereences in Methods).<br>Light microscopy data was acquired using Nikon Elements 5.1 (referenced in Methods).
Data analysis	Cryo-ET data processing (all available and referenced in Methods): IMOD v4.11.4 (open source, cited), Amira-Avizo 2020.2 (Thermo Fisher Scientific), Dynamo 1.1.454 (open source, cited), MATLAB 2019b (MathWorks).<br>Light Microscopy: FIJI (open source, cited), Huygens Essentials (SVI) 19.1. All particle tracking was done with the Trackmate plugin within FIJI (open source, cited), then analyzed in MATLAB 2019b using MSDanalyzer 1.1 (open source, cited) and Morphometrics 1.1.02.<br>KymographClear 2.0 and KymographDirect 2.1.<br>Codes are available (GitHub): https://github.com/NavarroVettiger/Navarro-et-al_2022

For manuscripts utilizing custom algorithms or software that are central to the research but not yet described in published literature, software must be made available to editors and reviewers. We strongly encourage code deposition in a community repository (e.g. GitHub). See the Nature Portfolio guidelines for submitting code & software for further information.

Data

Policy information about availability of data

All manuscripts must include a data availability statement. This statement should provide the following information, where applicable:

- Accession codes, unique identifiers, or web links for publicly available datasets
- A description of any restrictions on data availability
- For clinical datasets or third party data, please ensure that the statement adheres to our policy

Cryo-ET data: Representative tomograms deposited in EMDB: EMD-27479 (wild-type), EMD-27484 (ftsN-ΔSPOR), EMD-27485 (ΔenvC and ΔenvC ΔnlpD), EMD-27486 (ftsL*). Corresponding raw movie frames and stacks of tilt-series deposited as EMPIAR-11090 (wild-type), EMPIAR-11087 (ftsN-ΔSPOR), EMPIAR-11089 (ΔenvC and ΔenvC ΔnlpD) and EMPIAR-11088 (ftsL*). Data will be available upon publication of the manuscript in Nature Microbiology.
Light microscopy: key data to support the consclusions of the manuscript are shown in the main figures and Extended Data figures (Source Data. Additional substantiatin data, including raw data for all figures, are available upon request. Image analysis scripts are provided on GitHub: https://github.com/NavarroVettiger/Navarro-et-al_2022

Human research participants

Policy information about studies involving human research participants and Sex and Gender in Research.

Reporting on sex and gender	*Use the terms sex (biological attribute) and gender (shaped by social and cultural circumstances) carefully in order to avoid confusing both terms. Indicate if findings apply to only one sex or gender; describe whether sex and gender were considered in study design whether sex and/or gender was determined based on self-reporting or assigned and methods used. Provide in the source data disaggregated sex and gender data where this information has been collected, and consent has been obtained for sharing of individual-level data; provide overall numbers in this Reporting Summary. Please state if this information has not been collected. Report sex- and gender-based analyses where performed, justify reasons for lack of sex- and gender-based analysis.*
Population characteristics	*Describe the covariate-relevant population characteristics of the human research participants (e.g. age, genotypic information, past and current diagnosis and treatment categories). If you filled out the behavioural & social sciences study design questions and have nothing to add here, write "See above."*
Recruitment	*Describe how participants were recruited. Outline any potential self-selection bias or other biases that may be present and how these are likely to impact results.*
Ethics oversight	*Identify the organization(s) that approved the study protocol.*

Note that full information on the approval of the study protocol must also be provided in the manuscript.

Field-specific reporting

Please select the one below that is the best fit for your research. If you are not sure, read the appropriate sections before making your selection.

☒ Life sciences ☐ Behavioural & social sciences ☐ Ecological, evolutionary & environmental sciences

For a reference copy of the document with all sections, see nature.com/documents/nr-reporting-summary-flat.pdf

Life sciences study design

All studies must disclose on these points even when the disclosure is negative.

Sample size	Sample sizes were determined by available cryo-electron microscopy and cryo-FIB instrument time. A total of 109 tomogram were acquired (see Methods and Extended Data).<br>For light microscopy experiments no specific sample size calculation was made, since in general large number of cells were analyzed (965 cells for morphology quantification, ≥1000 cells in cell wall labeling experiments, ≥ 2000 single MreB trajectories etc.) over three independent biological replicates. Even when few cells (e.g. N ≤ 100) were analyzed for measuring constriction rates due to the slow nature of this process, significant differences among groups were detected.
Data exclusions	For cryo-ET, tilt-series exhibiting errors during data collection were excluded. Exclusion of error-containing or incomplete tilt-series is a standard, pre-established practice for cryo-ET processing.<br>For light microscopy data were excluded based on criteria described in Methods: 15-20 % of kymographs were excluded from analysis due to cell movement during constriction, 2.5 % of vertically imaged cells were trapped tilted and removed from circularity analysis. Particle tracking was limited to tracks consisting of ≥ 4 spots and log-log fits of MSD were calculated for tracks with R-square ≥ 0.95.
Replication	Tomograms were acquired from multiple cells, grid replicates (at least 5 grids per strain) were possible and come from cells vitrified on different days and from different batch cultures.<br>All light microscopy experiment were successfully repeated over three biological replicates.

Randomization	We did not randomize any of our data, as after data collection, all measurements and analysis were performed the same over all conditions. Cells for cryo-FIB and tilt-series collection were chosen at random on each TEM autogrid.<br>For light micrscopy, sample were imaged in random intervals. For live-cell imaging, cells one field of view from the edge of the agarose pad were imaged.
Blinding	It was not possible to blind any of our data during acquisition and analysis, since the cell morphology of the strains analyzed in this study is fundamentally different. Image processing and analysis procedures were carried out mostly computer-based using unbiased automated procedures.

Reporting for specific materials, systems and methods

We require information from authors about some types of materials, experimental systems and methods used in many studies. Here, indicate whether each material, system or method listed is relevant to your study. If you are not sure if a list item applies to your research, read the appropriate section before selecting a response.

Materials & experimental systems

n/a	Involved in the study
☒	☐ Antibodies
☒	☐ Eukaryotic cell lines
☒	☐ Palaeontology and archaeology
☒	☐ Animals and other organisms
☒	☐ Clinical data
☒	☐ Dual use research of concern

Methods

n/a	Involved in the study
☒	☐ ChIP-seq
☒	☐ Flow cytometry
☒	☐ MRI-based neuroimaging
